# The ATPase mechanism of UvrA_2_ reveals the distinct roles of proximal and distal ATPase sites in nucleotide excision repair

**DOI:** 10.1093/nar/gkz180

**Published:** 2019-03-20

**Authors:** Brandon C Case, Silas Hartley, Memie Osuga, David Jeruzalmi, Manju M Hingorani

**Affiliations:** 1 Department of Molecular Biology and Biochemistry, Wesleyan University, Middletown, CT 06459, USA; 2 Department of Chemistry and Biochemistry, City College of New York of the City University of New York, New York, NY 10031, USA; 3 Ph.D. Program in Biochemistry, The Graduate Center of the City University of New York, New York, NY 10016, USA; 4 Hunter College High School, New York, NY 10128, USA; 5 Ph.D. Programs in Chemistry and Biology, The Graduate Center of the City University of New York, New York, NY 10016, USA

## Abstract

The UvrA_2_ dimer finds lesions in DNA and initiates nucleotide excision repair. Each UvrA monomer contains two essential ATPase sites: proximal (P) and distal (D). The manner whereby their activities enable UvrA_2_ damage sensing and response remains to be clarified. We report three key findings from the first pre-steady state kinetic analysis of each site. Absent DNA, a P_2ATP_-D_2ADP_ species accumulates when the low-affinity proximal sites bind ATP and enable rapid ATP hydrolysis and phosphate release by the high-affinity distal sites, and ADP release limits catalytic turnover. Native DNA stimulates ATP hydrolysis by all four sites, causing UvrA_2_ to transition through a different species, P_2ADP_-D_2ADP_. Lesion-containing DNA changes the mechanism again, suppressing ATP hydrolysis by the proximal sites while distal sites cycle through hydrolysis and ADP release, to populate proximal ATP-bound species, P_2ATP_-D_empty_ and P_2ATP_-D_2ATP_. Thus, damaged and native DNA trigger distinct ATPase site activities, which could explain why UvrA_2_ forms stable complexes with UvrB on damaged DNA compared with weaker, more dynamic complexes on native DNA. Such specific coupling between the DNA substrate and the ATPase mechanism of each site provides new insights into how UvrA_2_ utilizes ATP for lesion search, recognition and repair.

## INTRODUCTION

**Figure 1.**
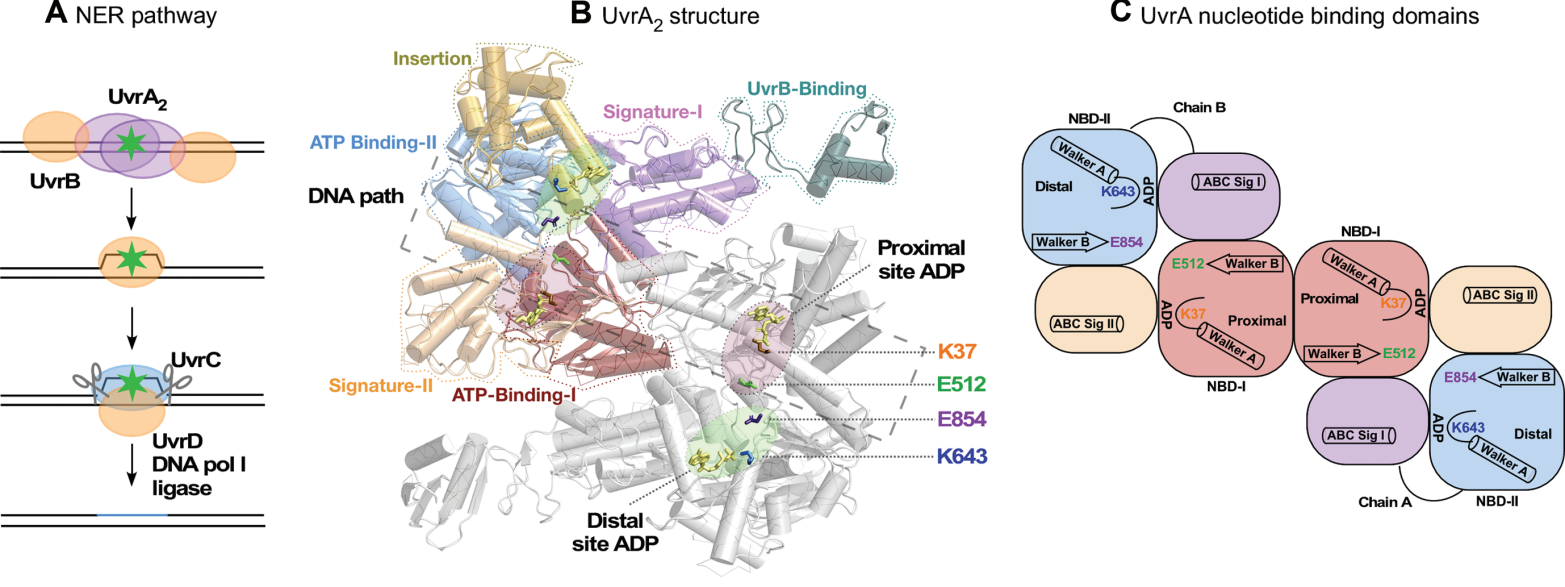
NER model and UvrA_2_ dimer showing two ATPase sites on each subunit. (**A**) Minimal NER pathway depicting lesion recognition by UvrA_2_, verification by UvrB, nicking of damaged DNA by UvrC and removal by UvrD, followed by DNA resynthesis and ligation by polymerase and ligase. (**B**) *Geobacillus stearothermophilus* UvrA_2_ structure with one subunit colored by domains and the other in gray. The composite proximal (pink circles) and distal (green circles) ATPase sites made by ATP-binding domain I/signature domain II and ATP-binding domain II/signature domain I, respectively, are labeled on the gray subunit, as are Walker A and B residues in each site. The bound ADP is depicted as sticks (yellow) and the DNA binding groove by a dashed line (PDB code: 2R6F) (26). (**C**) Schematic of the composite ATPase sites, showing the two nucleotide-binding domains (NBD) in each subunit (chains A, B).

Nucleotide excision repair (NER) processes diverse lesions in DNA damaged by chemical modification (e.g. benzo[*a*]pyrene adducts) or UV radiation (e.g. cyclobutane pyrimidine dimers) (1–4). This multi-step pathway employs different proteins to scan the genome, distinguish damaged from undamaged (native) DNA, incise and remove the lesion-containing section of single-stranded DNA and, finally, mediate DNA synthesis using the undamaged strand as template (Figure 1A). Given the importance of NER for maintaining genome integrity, it is not surprising that these protein functions have been conserved through evolution, although, interestingly, the proteins themselves have not. Defects in eukaryotic NER are associated with cancer predisposition, UV sensitivity and premature aging among other conditions related to genome instability.

In bacteria, NER is initiated by UvrA_2_, which scans dsDNA and binds to lesions with high affinity (5–8) (Figure 1A). Once a lesion is located, the DNA is handed off to UvrB, a helicase that translocates along single-stranded DNA and verifies the damage via contact with a β-hairpin (9–14). The timing and context of interaction between UvrA_2_ and UvrB is still under investigation. Biochemical and structural studies show that a UvrA_2_B_2_ complex can form without DNA (15–17), and single molecule studies implicate this complex in the initial search (6), but also indicate that UvrA_2_ can find a lesion by itself and then recruit UvrB (5). The handoff to UvrB is accompanied by expulsion of UvrA_2_ from the damage-sensing complex (18). Lesion-bound UvrB recruits UvrC, a dual 5′ and 3′ nuclease, to nick the damaged strand at sites flanking the lesion (19–22). Subsequent strand displacement by UvrD helicase allows gap filling by DNA polymerase I, and finally DNA ligase completes repair (1).

During NER, UvrA_2_ binds DNA in a groove along the dimer interface, and has been captured in broadly defined ‘open’ and ‘closed’ conformations in crystal structures (15,23–26). Figure 1B shows the structure of *Geobacillus stearothermophilus* UvrA_2_ in an open conformation (26). It has been proposed that transient switching between these conformations enables UvrA_2_ to scan DNA for lesions (15). UvrA_2_ is also an ATPase from the ATP-binding cassette (ABC) family of proteins, which have a distinctive composite nucleotide-binding site for coupling ATP binding and hydrolysis to conformational changes (Figure 1C) (27–31). UvrA_2_ is an unusual member of this family in that it has two ATPase sites per monomer instead of one, resulting in a total of four sites on the dimer (26,32). The two sites on each monomer are termed ‘proximal’ and ‘distal’, and each site comprises an ATP-binding domain with Walker A and B motifs and a signature domain with the ABC signature motif. The proximal site, which lies closer to the DNA-binding groove at the dimer interface, is formed by ATP-binding domain I and signature domain II (which also has residues critical for interaction with DNA and with UvrB) (15). The distal site is formed by ATP-binding domain II and signature domain I (which has the UvrB-binding domain and DNA-binding insertion domain) (Figure 1B and C).

Previous research on how UvrA_2_ utilizes ATP has shown that both proximal and distal ATPase sites are required for its function (25,32–34). Mutants of conserved Walker A and B motif residues have been employed to parse the role of each ATPase site; specifically, a Walker A lysine that hydrogen bonds with the β-phosphate and is important for nucleotide binding, and a Walker B glutamate that serves as a general base to activate water for ATP hydrolysis (31,35,36). Some key findings from these studies are: UvrA_2_ dimer is more stable in the presence of ATP (37,38), possibly as a mixed nucleotide-bound/free species in which the proximal site is empty and the distal site may be occupied by ATP or ADP (5,33,39); ATP promotes UvrA_2_ interaction with UvrB and its recruitment to the damage site, again possibly as a mixed species in which the distal site is ATP-bound (5,33,40); and, ATP hydrolysis facilitates UvrA_2_ dissociation from DNA (34). Based on structural, biochemical and *in vivo* single-molecule imaging data, it has been proposed that the proximal site is involved in regulating interactions among UvrA_2_, UvrB and DNA, and the distal site is involved in genome scanning (5,15). However, some studies have yielded discordant results; for example, the ATPase site mutants have been reported to suffer complete loss of ATP binding and hydrolysis or to maintain residual activity, which confounds interpretation of their function, and have been found either proficient or deficient in specific binding to lesions (5,32–34). These discrepancies, which could be due to differences in assays, reaction conditions and/or nucleotide or ATPase contaminants in the protein preparations, have hindered definitive understanding of the role of UvrA_2_ ATPase activity in NER. More importantly, the stoichiometry and kinetics of ATP binding and hydrolysis catalyzed by UvrA_2_ have not been determined thus far. Due to this fundamental gap in knowledge, questions about the proximal and distal site ATPase mechanisms, especially how they are coupled to each other and to UvrA_2_ interactions with DNA and UvrB, remain to be resolved.

In this study, we measured the rates of distinct steps in the ATPase reaction catalyzed by each site on UvrA_2_, from ATP binding to hydrolysis and product release, in the absence and presence of native and lesion-containing DNA. We chose *G. stearothermophilus* UvrA_2_ as the model protein, since crystal structures of this protein in different states are available to help interpret the kinetic data (15,26,41,42). We also leveraged information from the highest resolution UvrA_2_ structure (2.0 Å), reported here for the first time (*Thermotoga maritima* UvrA_2_; PDB code: 6N9L). Before initiating mechanistic analysis, we addressed the problem of potential nucleotide contamination by testing *G. stearothermophilus* UvrA_2_ purified by a previously reported protocol (26). The results of a luciferase-based assay for ADP and ATP showed that the protein co-purifies with about one ADP bound per UvrA_2_. Adjustment of the purification protocol includes mild heat treatment that successfully removed all the ADP (Supplementary Figure S1). We also prepared a double Walker A mutant with the conserved lysines in both sites (K37 and K643) mutated to alanine, in order to detect any contaminating ATPases against a background of catalytically inactive UvrA_2_. ATPase assays with ^K37A-K643A^UvrA_2_ found no detectable activity (Figure 6A). Pre-steady state kinetic analysis of wild-type and mutant UvrA_2_ proteins identified stark asymmetry in the ATP binding and hydrolysis activities of the proximal and distal sites, and showed that they are modulated differentially by native and lesion-containing DNA. The findings offer new ideas for understanding how UvrA_2_ utilizes ATP to discriminate between native versus damaged DNA and initiate NER.

## MATERIALS AND METHODS

### Proteins, DNA and other reagents

Wild-type *G. stearothermophilus* UvrA_2_ was overexpressed in *Escherichia coli* Rosetta (DE3) pLysS cells (Millipore Sigma) from pET28a-NHis-UvrA plasmid and purified by a modified version of a previously described procedure (15). Briefly, cells were grown from a fresh colony in LB media at 37°C to 0.6 OD_600_, induced with 1 mM isopropyl β-D-1-thiogalactopyranoside (IPTG) for 3 h at 30°C and pelleted by centrifugation (all steps after growth were at 4°C unless noted otherwise). The cell pellet was resuspended and lysed by homogenization in buffer (50 mM Tris–HCl, pH 8, 0.5 M NaCl, 20% sucrose) containing a protease inhibitor cocktail and 1 mM phenylmethylsulfonyl fluoride (PMSF) (Millipore Sigma). To remove DNA and nucleotide contaminants, the lysate was warmed at 55°C for 15 min and then cooled to 4°C, clarified by ultracentrifugation (50 000 *g*), brought to 1 M NaCl and treated with 0.5% polyethyleneimine (PEI), and then clarified again by centrifugation (25 000 *g*). Next, protein was precipitated overnight with 65% ammonium sulfate (43) and the pellet was suspended in buffer (50 mM Tris–HCl, pH 8, 0.5 M NaCl, 10 mM imidazole, 5 mM β-mercaptoethanol) for column chromatography. The protein solution was purified over a Ni-NTA agarose column (Qiagen) using a 10–150 mM imidazole gradient in the same buffer. Peak fractions were pooled, diluted with buffer (50 mM Tris–HCl, pH 7.4, 5 mM β-mercaptoethanol) to 0.2 M NaCl and further purified on a Heparin Sepharose 6 column (GE Healthcare) using a 0.2–1 M NaCl gradient in the same buffer. Finally, peak fractions were pooled, the protein was dialyzed against buffer (25 mM Tris–HCl, pH 7.4, 0.25 M NaCl, 20% glycerol, 1 mM DTT), and aliquots were flash frozen in liquid nitrogen for storage at −80°C (freshly thawed aliquots were used for each experiment). Preparation of overexpression clones for UvrA_2_ Walker A mutants, ^K37A^UvrA_2_, ^K643A^UvrA_2_ and ^K37A-K643A^UvrA_2_, has been described previously (15), and clones for Walker B mutants, ^E512A^UvrA_2_ and ^E854A^UvrA_2_, were prepared using the QuikChange Lightning kit (Agilent Technologies); primer sequences: E512A forward: 5′-CGT GCT CGA CGC GCC GTC GAT CGG-3′; E512A reverse: 5′-CCG ATC GAC GGC GCG TCG AGC ACG-3′; E854A forward: 5′-GCT CTA CAT TTT GGA CGC GCC GAC GAC C-3′; E854A reverse: 5′-GGT CGT CGG CGC GTC CAA AAT GTA GAG C-3′. All mutant proteins were purified as described for wild-type UvrA_2_ above. Protein samples were run on an agarose gel and stained with ethidium bromide to test for DNA contamination (none detected) and by a luciferase-based bioluminescence kit (Millipore Sigma) for nucleotide contamination (no significant level of ADP or ATP was detected in proteins purified by the above protocol; see Supplementary Data for method and results, Figure S1). *E. coli* phosphate binding protein (PBP) was purified and labeled with MDCC as described (44). Preparation and crystallization of *T. maritima* UvrA Δ117-399 is described in Supplementary Data.

All DNAs were purchased from Integrated DNA Technologies. Unlabeled DNA strands were purified in-house by electrophoresis in 6 M urea/18% (w/v) polyacrylamide gels followed by electroelution and ethanol precipitation, and fluorescein-labeled strands were obtained HPLC purified and desalted. The sequences are: template: 5′-TGG ATT ACT TAC GGC CAC ATT ACT ACT GGA ACT CAG AAC GAG CTG ACA GG-3′ (unlabeled for ATPase assays with native DNA and 5′ end-labeled with 6-FAM for native DNA binding assays); native complement: 5′-CCT GTC AGC TCG TTC TGA GTT CCA GTA GTA ATG TGG CCG TAA GTA ATC CA-3′; fluorescein lesion complement: 5′-CCT GTC AGC TCG TTC TGA GTT CCA G/iFluorT/A GTA ATG TGG CCG TAA GTA ATC CA-3′. Duplex DNA substrates were prepared by annealing complementary strands in 1:1 ratio by heating for 1 min at 95°C followed by slow cooling O/N to room temperature in buffer (20 mM Tris–HCl, pH 7.4, 0.1 M NaCl), and tested by non-denaturing PAGE to confirm >95% duplex. DNA binding to UvrA_2_ was measured by change in fluorescence anisotropy of fluorescein end-labeled (undamaged) or internally labeled (damaged) DNAs (see Supplementary Data for method and results, Figure S6). All nucleotides, ATP, ADP, mant-ATP and mant-ADP, were purchased from Millipore Sigma.

### Mant-nucleotide binding assays

Mant-ATP and mant-ADP binding kinetics were measured by monitoring change in fluorescence of the mant fluorophore (*λ*_EX_ = 352 nm, *λ*_EM_ > 420 nm) over time when increasing concentrations of the nucleotide were mixed with wild-type or mutant UvrA_2_ in the absence or presence of DNA in a stopped-flow instrument (KinTek Corp, Austin TX) in buffer (20 mM Tris–HCl, pH 7.4, 0.15 M NaCl, 5% glycerol, 5 mM MgCl_2_, 5 mM DTT) at 40°C (final concentrations: 0.1 μM UvrA_2_, ± 0.2 μM DNA and 2.5–10 μM mant-ATP or mant-ADP). Mant-ADP dissociation kinetics were measured directly by monitoring the change in fluorescence over time on mixing wild-type or mutant UvrA_2_ pre-incubated with mant-ADP, in the absence or presence of DNA, with excess unlabeled ADP or ATP on the stopped-flow (final concentrations: 0.1 μM UvrA_2_, ± 0.2 μM DNA, 10 μM mant-ADP and 2 mM ADP or 2 mM ATP). The signal from 3 to 5 traces was averaged for each experiment and corrected for background fluorescence from mant-nucleotide alone (mant-ADP photobleaching caused a slow, linear decrease in signal to 15% at most over the 150-s dissociation time scale). Association data were fit to a single exponential equation to determine the observed rate (*k*_obs_), and linear dependence of this rate versus nucleotide concentration yielded the bimolecular rate constant, *k*_on_ and the dissociation rate, *k*_off_ (*k*_obs_ = *k*_on_[mant-nucleotide] + *k*_off_). Error bars report standard error of the mean (S.E.M.) from *N* = 3. Dissociation data were fit to a single exponential equation to determine *k*_off_.

The stoichiometry of nucleotide binding was measured by Förster resonance energy transfer (FRET) between UvrA_2_ tryptophans (donor) and mant-ADP (acceptor). UvrA_2_ (3 μM) was titrated with mant-ADP (0–20 μM) in buffer (20 mM Tris–HCl, pH 7.4, 0.15 M NaCl, 5% glycerol, 5 mM MgCl_2_, 5 mM DTT) at 40°C, and fluorescence intensity was measured after mixing and a 1-min incubation (*λ*_EX_ = 290 nm, *λ*_EM_ = 305–400 nm; Jobin-Yvon Horiba Fluoromax-3). Emission spectra were collected for UvrA_2_ alone (D), mant-ADP alone (A) and UvrA_2_ plus mant-ADP (DA), and the spectra were integrated using Grams/AI software (Thermo Scientific); the fluorescence intensity of A was subtracted from that of DA to correct for background mant-ADP excitation at 290 nm, and fluorescence intensity of D was aligned with the initial value for DA (at zero mant-ADP). FRET efficiency was calculated as ${E_{{\rm{FRET}}}} = \ 1 - \left( {\frac{{{F_{{\rm{DA}}}}}}{{{F_{\rm{D}}}}}} \right)$; *F*_DA_ and *F*_D_ are the corrected fluorescence intensities of DA and D, respectively. *E*_FRET_ values from three independent experiments were averaged and plotted versus mant-ADP concentration. The inflection point between initial and final slopes of the isotherm yielded the stoichiometry of mant-ADP binding to UvrA_2_ (error bars report S.E.M. from *N* = 3).

### Phosphate release assays

Phosphate (Pi) release from UvrA_2_ after ATP hydrolysis was measured under pre-steady state conditions by monitoring change in fluorescence of the MDCC fluorophore (*λ*_EX_ = 425 nm, *λ*_EM_ > 450 nm) when wild-type or mutant UvrA_2_ and ^MDCC^PBP, in the absence or presence of DNA, were mixed with ATP on a stopped flow instrument in buffer (20 mM Tris–HCl, pH 7.4, 0.15 M NaCl, 5% glycerol, 5 mM MgCl_2_, 5 mM DTT) containing a Pi contaminant mopping system of 0.1 unit/mL polynucleotide phosphorylase (Millipore Sigma) and 0.2 mM 7-methylguanosine (R.I. Chemical Inc., Orange, CA) at 40°C (final concentrations: 0.125–2 μM UvrA_2_ and 1 mM ATP, or 2 μM UvrA_2_ and 10 μM to 2 mM ATP, or 0.25 μM UvrA_2_, ± 1 μM DNA and 1 mM ATP, and 15 μM ^MDCC^PBP) (45). The signal from 4 to 6 traces was averaged for each experiment, converted to Pi concentration using a calibration curve generated with standard Pi solution (Millipore Sigma) under the same conditions (Supplementary Figure S4A), and corrected for a low background signal at zero time. The data were fit to a double exponential + linear equation (${A_1}{e^{ - {k_1}t}} + {A_2}{e^{ - {k_2}t}} + {k_3}t$; kinetic traces with lag, burst and linear phases; Supplementary Figure S4D) or linear equation (linear kinetic traces) for initial estimation of the burst and steady state rates (*k*_cat_ = linear slope/4 sites × [UvrA_2_] for wild-type or slope/2 sites × [UvrA_2_] for mutants). Data from the concentration series were fit simultaneously using KinTek and FitSpace Explorer (46–48) to determine a minimal kinetic mechanism. The raw data, together with details of model development and data fitting by KinTek Explorer, are available as Supplementary Data at NAR Online.

## RESULTS

Transient kinetic measurements of wild-type UvrA_2_, as well as proximal and distal Walker A and B mutants, were performed to detail the ATP binding, hydrolysis and product release mechanisms of these sites. The ATPase kinetics in the absence and presence of DNA reveal specific coupling between each site and type of DNA, which could help explain the different actions of UvrA_2_ on native DNA (search for lesions) and at a damage lesion (signal repair).

### Asymmetric nucleotide binding to proximal (weak) and distal (tight) ATPase sites on the UvrA_2_ dimer

**Figure 2.**
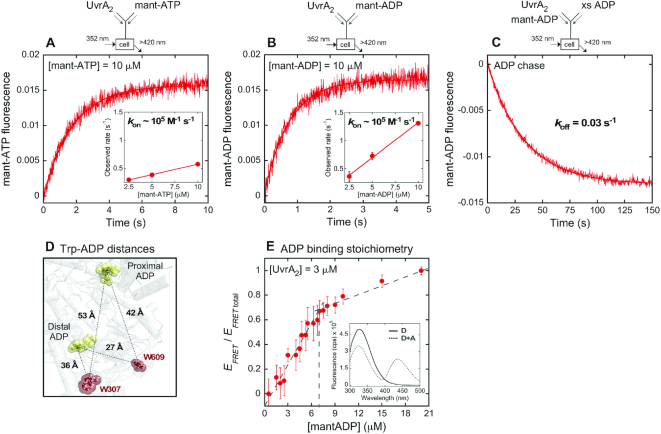
Kinetics, affinity and stoichiometry of nucleotide binding to UvrA_2_. Nucleotide binding was monitored by increase in fluorescence on mixing UvrA_2_ with (**A**) mant-ATP or (**B**) mant-ADP (final: 0.1 μM UvrA_2_ and 2.5–10 μM mant-nucleotide; 10 μM trace shown here; see Supplementary Figure S2). Time traces fit to a single exponential yield rates that depend linearly on nucleotide concentration, and the slope yields *k*_on_ = 0.4 and 1.3 × 10^5^ M^−1^ s^−1^ for mant-ATP and mant-ADP, respectively (A and B, inset). (**C**) Nucleotide dissociation was measured by pre-incubating mant-ADP with UvrA_2_ and mixing with excess unlabeled ADP chase (final: 0.1 μM UvrA_2_, 10 μM mant-ADP and 2 mM ADP). The decrease in fluorescence fit to a single exponential yields *k*_off_ = 0.03 ± 0.0002 s^−1^ (*k*_off_/*k*_on_ yields a tight *K*_D1_ = 0.23 μM for mant-ADP; Table 1). (**D**) UvrA_2_ structure showing distances between tryptophans W307, W609 (donor) and nucleotides (acceptor mant-ADP) (PDB code: 2R6F). (**E**) Nucleotide binding stoichiometry was measured by FRET on titrating UvrA_2_ (3 μM) with increasing mant-ADP; error bars report S.E.M. (*N* = 3). The inset shows spectra for UvrA_2_ alone (donor, D) and with mant-ADP (donor + acceptor, D+A). The binding isotherm has an inflection point of 6.8 μM (2.3 mant-ADP per UvrA_2_ dimer).

We used ATP and ADP analogs with the ribose modified by 2′(3′)-*O*-(*N*-methylanthraniloyl) fluorophore (mant) (49) to measure the kinetics and stoichiometry of nucleotide binding to UvrA_2_. Mant-nucleotide fluorescence intensity increases on binding to UvrA_2_, as reported previously for other ATPases (50,51). Monitoring the signal over time after mixing 0.1 μM UvrA_2_ with 10 μM mant-ATP on a stopped flow yields a binding rate of 0.6 s^−1^ (Figure 2A; kinetic trace under pseudo first order conditions fit with a single exponential function). A titration with mant-ATP reveals linear concentration dependence of the binding rate, providing a bimolecular association rate constant from the slope and a rough estimate of the dissociation rate from the *Y*-intercept (*k*_on_ = 0.4 × 10^5^ M^−1^ s^−1^, *k*_off(estimate)_ = 0.2 s^−1^; Figure 2A inset; Supplementary Figures S2B, S2D, Table 1). A similar analysis of mant-ADP binding kinetics yields comparable rates (*k*_on_ = 1.3 × 10^5^ M^−1^ s^−1^, *k*_off(estimate)_ = 0.06 s^−1^; Figure 2B, Supplementary Figures S2C, S2E, Table 1). The dissociation rate was also measured directly by pre-incubating mant-ADP with UvrA_2_ and mixing with excess unlabeled ADP to prevent mant-ADP rebinding. In this experiment, mant-ADP fluorescence decreases as a single exponential over time and yields a slow *k*_off_ = 0.03 s^−1^ (Figure 2C). The *k*_off_/*k*_on_ ratio yields a dissociation constant of 0.23 μM for mant-ADP, indicating a high affinity and stable interaction with UvrA_2_ (*K*_D1_; Table 1). Mant-ATP dissociation was not measured directly, as this nucleotide is hydrolyzed in the time frame of the experiment (from the mant-ATP *k*_off_ estimate above, *K*_D1_ ∼5 μM).

**Table 1.** Measured parameters for the UvrA_2_ ATPase mechanism

UvrA_2_	DNA	mantATP *k*_on_ (× 10^5^ M^−1^ s^−1^)	mantADP *k*_on_ (× 10^5^ M^−1^ s^−1^)	mantADP *k*_off_ (ADP chase; s^−1^)	mantADP *K*_D1_ (μM)	mantADP *k*_off_ (ATP chase; s^−1^)	*k*_cat_(s^−1^)
Wild Type	None	^a^0.4	1.3	0.03	0.2	2.3	^b^0.2
	Native	0.6	1.6	0.04	0.3	8.7	0.8
	Lesion	0.6	1.7	0.05	0.3	13	1.4
K37A	None	0.5	2.3	0.05	0.2	0.06	0.04
	Native	0.8	2.2	0.1	0.4	0.1	0.04
	Lesion	0.7	2.2	0.1	0.5	0.1	0.03
E512A	None	0.5	2.6	0.05	0.2	5	0.3
	Native	0.6	2.6	0.1	0.4	15	0.6
	Lesion	0.7	2	0.1	0.6	25	2
K643A	None	^c^ND	ND	ND	ND	ND	0.1
	Native	ND	ND	ND	ND	ND	1.4
	Lesion	ND	ND	ND	ND	ND	0.3
E854A	None	0.9	4	0.07	0.2	4	0.6
	Native	1	3.3	0.1	0.4	7	0.3
	Lesion	0.9	3.2	0.2	0.5	13	0.3

^a^S.E.M. range from 2 to 10% for all reported values from 2 to 4 independent measurements.

^b^ *k*_cat_ = linear slope/4 sites × [UvrA_2_] for wild-type, and slope/2 sites × [UvrA_2_] for mutants.

^c^ND = not detectable.

The next step was to determine how many of the four ATPase sites on UvrA_2_ bind nucleotides. We measured the stoichiometry by titrating UvrA_2_ (3 μM) at a concentration well above the measured dissociation constant (0.23 μM) with increasing amounts of mant-ADP. In this case, the reporter was UvrA_2_ tryptophan fluorescence quenching due to FRET to mant-ADP (52). Figure 2D shows distances between potential tryptophan donors and mant-ADP acceptors at the proximal and distal sites in *G. stearothermophilus* UvrA_2_; tryptophan - mant *R*_0_ ∼25 Å (52,53). As shown in Figure 2E, FRET efficiency increases linearly with mant-ADP concentration until saturation, and the inflection point yields a ratio of 2.3 mant-ADP bound per UvrA_2_ (Figure 2E, inset shows the emission spectra of UvrA_2_, alone and with mant-ADP). These results indicate asymmetry in the nucleotide binding properties of UvrA_2_ since only two of the four ATPase sites bind ADP with high affinity.

**Figure 3.**
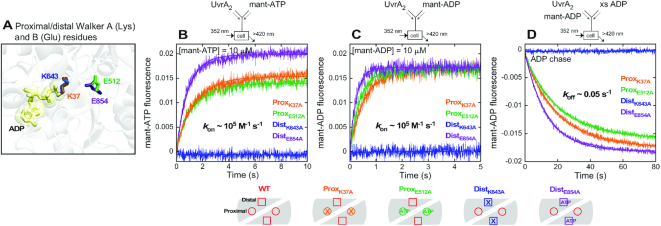
The distal ATPase sites on UvrA_2_ bind nucleotides with high affinity. (**A**) Aligned proximal and distal ATPase sites depicting Walker A and B residues (PDB code: 2R6F). Kinetics of (**B**) mant-ATP and (**C**) mant-ADP binding to Walker A (^K37A^UvrA_2_, ^K643A^UvrA_2_) and B (^E512A^UvrA_2_, ^E854A^UvrA_2_) mutants of the proximal and distal sites were measured as described for wild-type UvrA_2_ in Figure 2 (final: 0.1 μM UvrA_2_ and 2.5–10 μM mant-nucleotide; 10 μM trace shown; Supplementary Figure S2). All mutants yielded binding rate constants similar to wild-type, except for ^K643A^UvrA_2_, which did not bind either nucleotide (Table 1). (**D**) mant-ADP dissociation was measured as for wild-type in Figure 2 (final: 0.1 μM UvrA_2_, 10 μM mant-ADP and 2 mM ADP). All mutants had similar dissociation rates as wild-type, except for ^K643A^UvrA_2_, which did not bind mant-ADP (Table 1).

The above experiments do not reveal which two sites bind ADP tightly and whether asymmetry exists within a monomer (between each proximal and distal site) or between monomers (between the pairs of sites across the dimer) (Figure 1B and C). We addressed this question using ATPase mutants in which the conserved Walker A lysine was replaced with alanine to disrupt ATP binding (proximal: K37A; distal: K643A; Figure 3A) (54), and the conserved Walker B glutamate was replaced with alanine to disrupt ATP hydrolysis while (likely) retaining ATP binding (proximal: E512A; distal: E854A; Figure 3A) (36). The kinetics of ATP and ADP binding to the mutants was measured as for wild-type UvrA_2_ (Figure 2A). As shown in Figure 3B and C, both Walker B mutants, ^E512A^UvrA_2_ (proximal) and ^E854A^UvrA_2_ (distal), exhibit similar mant-ATP and mant-ADP binding kinetics as wild-type (Figure 2A), with *k*_on_ on the order of 10^5^ M^−1^ s^−1^. Thus, this mutation does not disrupt nucleotide binding to UvrA_2_ (Supplementary Figures S2D, S2E and Table 1); a slightly higher signal for mant-ATP-bound ^E854A^UvrA_2_ may indicate a change in the local environment of the nucleotide, although this difference is not observed with mant-ADP. The Walker A mutant ^K37A^UvrA_2_, in which proximal ATP binding is disrupted while the distal site is intact, also exhibits a similar nucleotide binding rate. In contrast, the complementary Walker A mutant ^K643A^UvrA_2_, in which distal ATP binding is disrupted while the proximal site is intact, shows no binding at nucleotide concentrations tested on the stopped flow (Figure 3B and C; Supplementary Figures S2D, S2E). According to these results, distal sites bind mant-nucleotides, but proximal sites do not. Mant-ADP dissociation measurements show that the three mutants, ^K37A^UvrA_2_, ^E512A^UvrA_2_ and ^E854A^UvrA_2_, bind mant-ADP with comparable high affinity and stability to wild-type (*k*_off_ ∼0.05 s^−1^, *K*_D_ ∼0.2 μM; Figure 3D, Table 1), whereas ^K643A^UvrA_2_ does not show any change in the baseline signal, consistent with its inability to bind mant-ADP under these conditions (Figure 3D). Moreover, the FRET-based assay used to measure mant-ADP binding stoichiometry (Figure 2E) reports weak interaction at best for ^K643A^UvrA_2_ (Supplementary Figure S2F). Together, these results clearly indicate that distal sites on UvrA_2_ bind ATP and ADP tightly, whereas proximal sites have weaker affinity. This finding is consistent with the detection of [α-^32^P]ATP bound to ^K37A^UvrA_2_ but not ^K643A^UvrA_2_ in a nitrocellulose filter binding assay (15). Thus, we can conclude that nucleotide binding asymmetry exists between the proximal and distal ATPase sites on each monomer in UvrA_2_, and the stoichiometry of two mant-ADP per UvrA_2_ reflects occupancy of the two high-affinity distal sites on the dimer (Figure 1B and C).

### Structural analysis of asymmetric nucleotide binding by UvrA_2_

To gain additional insights into the finding that nucleotides are held more tightly by the distal ATPase sites than proximal sites, we interrogated atomic models of several UvrA_2_ orthologs for underlying sources of the differential affinity. These included a newly determined high resolution 2.0 Å crystal structure of UvrA_2_ from *T. maritima* (Supplementary Figure S3A, Supplementary Table S1; PDB code: 6N9L) as well as published structures of *G. stearothermophilus, T. maritima, Deinococcus radiodurans* and *Mycobacterium tuberculosis* UvrA_2_ (15,23–26) (discussion of the new *T. maritima* UvrA_2_ structure is restricted to aspects relevant to this kinetic study; the reader is referred to the cited reports for detailed structural descriptions of UvrA_2_). Our analysis examined the number and types of interactions between the ATPase sites and the bound nucleotides, which was simplified by the high degree of sequence and structural conservation in both proximal and distal sites in these orthologs. Indeed, the eight ATPase sites in question could be easily superimposed using the Walker A sequence (Supplementary Table S2), highlighting the overall similarity in these domains among currently available structures. The largest deviation in the superpositions was found in the ABC signature and the Q-loop motifs.

**Figure 4.**
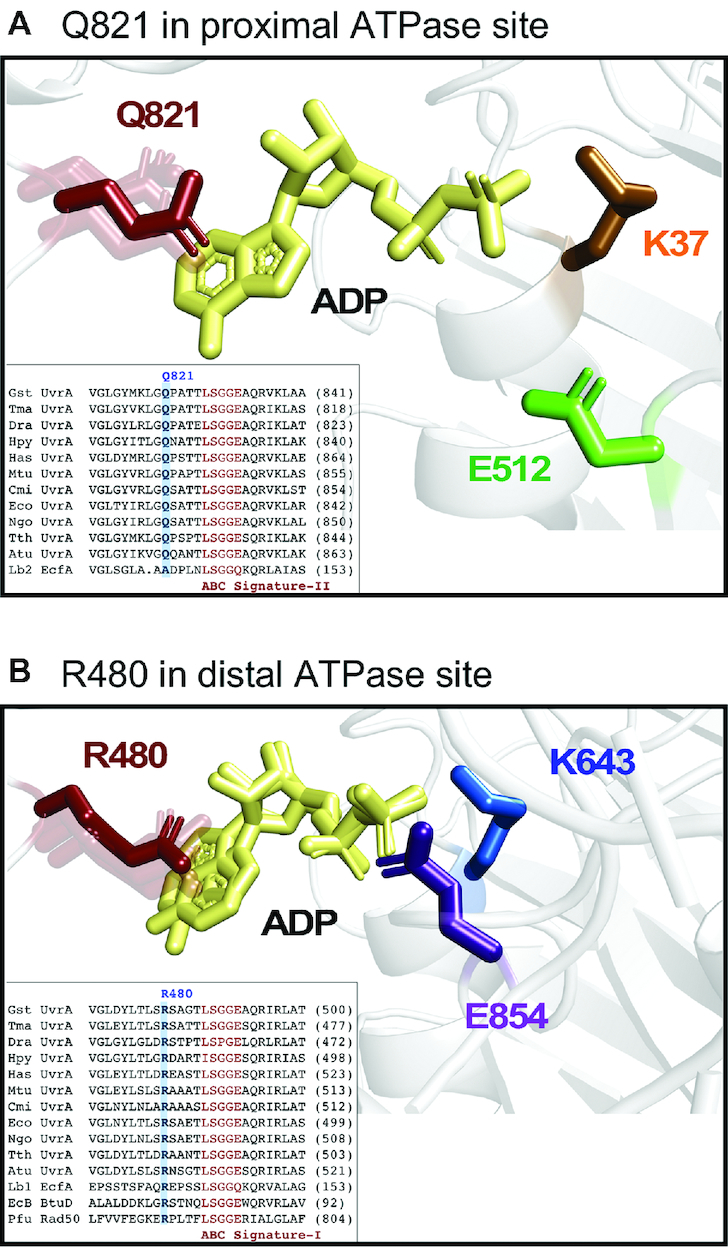
UvrA structures showing interactions of ADP with R480 and Q821 (*G. stearothermophilus* residue numbers). (**A**) Representation of the *T. maritima* proximal ATPase site showing K37, E512 and ADP, as well as Q821 highlighted in solid red among transparent Q821 residues from all other proximal ADP-bound UvrA structures (PDB ID: 2R6F, 2VF7 and 2VF8). (**B**) *T. maritima* distal ATPase site showing K643, E854 and ADP (ADP from 2R6F is also shown, demonstrating a ring flip observed in this site in some UvrA structures). *T. maritima* R480 is highlighted in solid red among transparent R480 residues from all other distal ADP-bound UvrA structures (PDB ID: 2R6F, 3UX8, 2VF7 and 2VF8) (15,25,26). The insets show sequence alignment of several UvrAs and ABC transporters. R480 and Q821 are highlighted in blue and the signature domain is shown in maroon. UvrAs: Gst – *G. stearothermophilus*, Tma – *T. maritima*, Dra – *D. radiodurans*, Hpy – *H. pylori*, Has – *H. salinarum*, Mtu – *M. tuberculosis*, Cmi – *C. michiganensis*, Eco – *E. coli*, Ngo – *N. gonorrhoeae*, Tth – *T. thermophilus*, Atu – *A. tumefaciens*; Transporters: Lb1/2 – (EcfA-A′) *L. brevis* chain A and chain C, EcB – (BtuD) *E. coli*; DNA damage repair protein: Pfu – (Rad50) *P. furiosus*.

Detailed examination of the complete set of contacts between UvrA_2_ and nucleotide at all the sites revealed 21 polar and 28 hydrophobic contacts per site, on average (both proximal and distal sites make 21 polar contacts, and 29 and 27 hydrophobic contacts, respectively). These contacts are highly conserved between the two types of sites and between orthologs, with one notable exception. The proximal site has a glutamine residue that precedes the ABC signature motif by five residues (Q821, *G. stearothermophilus* residue numbering; Figure 4A). In contrast, the distal ATPase site of every UvrA_2_ ortholog features an arginine in the equivalent position, which is involved in a pi–cation interaction with the aromatic base of adenine (R480; Figure 4B). While both residues stack on the adenine base, the more extensive interaction and pi–cation stacking by R480 implicate this residue in the higher affinity of distal sites for nucleotides. Both R480 and Q821 are absolutely conserved in a large primary sequence alignment of UvrA proteins (Figure 4 insets), and are located at the end of a region previously noted as the ‘structurally diverse region’ (SDR) in ABC importers (55,56).

To determine if our findings about the potential roles of the arginine and glutamine residues in UvrA generalize to the larger ABC family of ATPases, we interrogated the Protein Data Bank using the ScanProsite (ExPASy) tool and search patterns that include these residues in the SDR regions of UvrA: R-X(4)-L-S-G(2)-X and Q-X(4)-L-S-G(2)-X. The search revealed 13 entries containing both patterns, all of which were ABC ATPases. Three of these entries corresponded to a heterodimeric ABC ATPase, the ABCE1 RNase L inhibitor (PDB codes: 3J16, 4CRM and 5LL6), and the remaining 10 were other UvrA proteins. Separate searches with one or the other pattern revealed that the Energy-coupling factor ABC ATPase importer retained an arginine in a similar position to R480 at one nucleotide binding site and an alanine in a similar position to Q821 at the second site (PDB codes: 4HLU and 4ZIR) (57,58). It would be interesting to determine whether this difference confers asymmetry in nucleotide binding (and catalytic activity, as shown below) to other dimeric ABC ATPases, as observed with UvrA_2_.

### Asymmetric and linked ATPase activities of the proximal (slow) and distal (rapid burst) sites on UvrA_2_

The discovery of differential nucleotide binding by the proximal and distal sites raised the question whether these sites also hydrolyze ATP differentially. If true, this possibility has profound implications for understanding how UvrA_2_ utilizes the ATPase reaction to drive NER. We addressed this question by analyzing UvrA_2_-catalyzed ATP hydrolysis and phosphate (Pi) release under pre-steady state conditions, which can reveal the stoichiometry of ATP hydrolysis and rate-limiting steps in the mechanism. We used an assay developed by the Webb group in which rapid (10^8^ M^−1^ s^−1^) and high affinity (*K*_D_ = 0.1 μM) binding of Pi by phosphate-binding protein (PBP) labeled with 7-diethylamino-3-((((2-maleimidyl)ethyl)amino)carbonyl) coumarin (MDCC) leads to a large increase in MDCC fluorescence (44). Due to these properties, ^MDCC^PBP reports any free Pi in solution effectively at the rate at which it is released upon ATP hydrolysis by UvrA_2_, thus enabling transient kinetic measurements.

**Figure 5.**
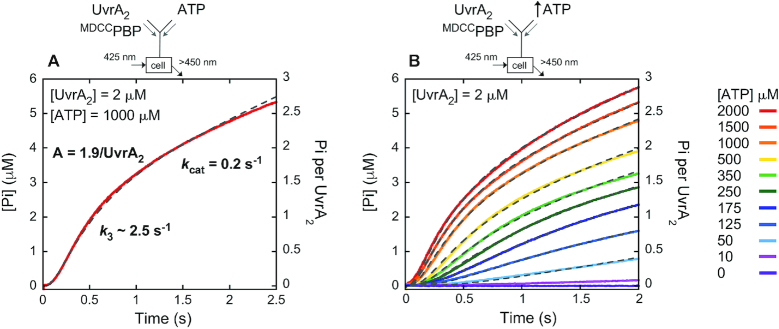
Two sites on UvrA_2_ catalyze a burst of ATP hydrolysis and phosphate (Pi) release. Pre-steady state kinetics of Pi release were measured by mixing UvrA_2_ and ^MDCC^PBP reporter with ATP at (**A**) increasing UvrA_2_ (final: 0.125–2 μM UvrA_2_, 1 mM ATP and 15 μM ^MDCC^PBP; 2 μM UvrA_2_ trace shown; Supplementary Figure S4) or (**B**) increasing ATP (final: 2 μM UvrA_2_, 10 μM to 2 mM ATP and 15 μM ^MDCC^PBP). After a slight lag, Pi is released rapidly in a burst phase followed by a slow linear phase that yields *k*_cat_ = 0.2 ± 0.03 s^−1^ (slope/4 × [UvrA_2_]; Table 1). Simultaneous fitting of data at all UvrA_2_ and ATP concentrations to a minimal ATPase mechanism (Scheme 1) yields *k*_3_ = 2.45 ± 0.04 s^−1^ that limits the burst rate, and a maximum burst amplitude *n* = 1.9 ± 0.03 ATP molecules hydrolyzed rapidly per UvrA_2_ per turnover (Table 2); gray dashed lines show the fit.

**Scheme 1.**
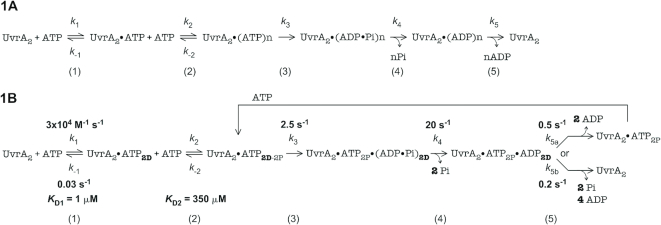
A minimal kinetic model of the UvrA_2_ ATPase mechanism. (**1A**) All ATPase kinetic data obtained for wild-type UvrA_2_ were fit to this model, with rate and affinity constants measured in mant-nucleotide binding/release and ATP hydrolysis/Pi release experiments serving as initial estimates for global fitting by KinTek Explorer (Supplementary Data). The best fit rate constants and stoichiometry are listed in Table 2 and shown in bold in (**1B**), which also includes ATPase site specific information from mutant analysis. The reaction starts with (1) rapid ATP binding by the high affinity distal sites (ATP_2D_), and then (2) the low affinity proximal sites (ATP_2D-2P_). A subsequent slow step (3) that we speculate is associated with ATP hydrolysis by the distal sites (ADP_2D_, Pi_2D_) is followed by (4) fast Pi release. Finally, a slow step involving (5a) ADP release from distal sites, or additionally (5b) ATP hydrolysis and product release by proximal sites, limits the catalytic turnover rate (*k*_cat_).

The reaction was initiated by mixing UvrA_2_ and ^MDCC^PBP with ATP in a stopped-flow apparatus and Pi formation was monitored over time (Figure 5A). The kinetic trace for 2 μM UvrA_2_ mixed with 1 mM ATP shows a slight lag phase and then a burst of Pi followed by a linear phase. The lag indicates at least two steps in the reaction leading to fast ATP hydrolysis and Pi release (which can be described by a double exponential function), and then a slow step limits steady state turnover (which can be described by a linear function) (59). Fitting the trace to a double exponential + linear function yields a rate of 2.4 s^−1^ for the burst, and the linear slope yields a *k*_cat_ of 0.2 s^−1^ (slope/4 sites × [UvrA_2_]); the same *k*_cat_ was obtained from steady state malachite green-based ATPase experiments (data not shown) (60). The burst amplitude is 3.4 μM, which represents the amount of ATP hydrolyzed rapidly by 2 μM UvrA_2_ in the first turnover. The lag of ∼100 ms preceding the burst indicates that a slow step(s) occurs before/at ATP hydrolysis as well and is followed by Pi release. This experiment was repeated at varying concentrations of UvrA_2_ and constant ATP (1 mM) to accurately determine the stoichiometry of ATP hydrolysis from the burst amplitude of the first turnover (Supplementary Figure S4B). In addition, complementary experiments were performed at constant UvrA_2_ (2 μM) and increasing ATP concentrations to determine how the different nucleotide binding affinities of proximal (weak) and distal (tight) sites impact ATPase activity. As shown in Figure 5B, the burst of Pi release increases with ATP concentration, approaching half-maximum at ∼350 μM and maximum at >1.5 mM. The need for such high ATP concentrations implies that the low affinity proximal sites play an important role in the ATPase activity of UvrA_2_. The data from all these experiments were analyzed by global fitting to a model ATPase mechanism, as shown in Scheme 1 and explained below.

### A minimal kinetic model for UvrA_2_ ATPase activity

The goal was to develop a kinetic mechanism with the minimal number of steps and parameters required to simultaneously fit all the ATPase data described above. The measured nucleotide binding (Figures 2 and 3) and ATPase rates (Figure 5 and Supplementary Figure S4B) were used as initial estimates and allowed to float during data fitting, as was the number of ATPase sites catalyzing the burst of hydrolysis. The raw data, together with details of model development and data fitting by KinTek Explorer, are available as Supplementary Data at NAR Online (46,47). The best fit model is shown in Scheme 1A, and the corresponding rate constants are shown in Table 2 and Scheme 1B, which also includes findings from experiments with UvrA_2_ mutants described below. The fits are shown as dashed lines overlaid on the corresponding experimental data in Figure 5A,B and Supplementary Figure S4B. The reaction begins with ATP binding rapidly to the two high affinity distal sites (step 1; *K*_D1_ = 1 μM; UvrA_2_•ATP_2D_) and two low affinity proximal sites (step 2; *K*_D2_ = 350 μM; UvrA_2_•ATP_2D-2P_). ATP binding is followed by a slow step that has been designated as ATP hydrolysis (step 3; *k*_3_ = 2.5 s^−1^), and then another step designated as Pi release (step 4; *k*_4_ = 20 s^−1^). The final step in the reaction, which limits the steady state turnover rate, is designated as ADP release (step 5; *k*_5a_ = 0.5 s^−1^ or *k*_5b_ = 0.2 s^−1^ depending on the number of active sites per turnover, as explained further below).

**Table 2.** Best fit parameters for the UvrA_2_ ATPase mechanism

Parameters	Best-fit values	Event
^a^ ***k*_1_**	*^b^* 3 ± 0.0003 × 10^4^ M^−1^ s^−1^*^c^* (2.8–3.3)	ATP binding (distal sites)
***k*_-1_**	0.03 s^−1^	ATP dissociation
*K*_D1_	1 μM	
***K*_D2_**	350 μM	ATP binding (proximal sites)
*k*_3_	2.45 ± 0.04 s^−1^ (2.2–2.9)	ATP hydrolysis
*k*_4_	19 ± 0.6 s^−1^ (14–24)	Pi release
*k*_5a_	0.44 ± 0.009 s^−1^ (0.38–0.52)	ADP release (two active sites/turnover)
*k*_5b_	0.18 ± 0.002 s^−1^ (0.16–0.21)	ADP release (four active sites/turnover)
*n*	1.9 ± 0.03	Burst ATPase sites

^a^Parameters in bold were linked during global fitting and confidence contour analysis.

^b^Standard errors are shown for parameters allowed to float during data fitting.

^c^Upper and lower limits for parameters from confidence contour analysis are in parentheses.

These are the minimal number of steps required to simultaneously fit all the ATPase data described above and obtain well constrained parameters for each step (error analysis by FitSpace Explorer is described in Supplementary Data, and the resulting limits on rate constants are shown in Table 2 and Supplementary Figure S4C) (48). The model mechanism shows that: (i) the burst rate is determined by at least two steps, which we propose are associated with ATP hydrolysis at 2.5 s^−1^ and subsequent Pi release at 20 s^−1^ (note that these two rates likely reflect slow protein conformational dynamics that enable relatively fast ATP hydrolysis and Pi release events); (ii) the best fit burst amplitude (*n*) of two ATP molecules per UvrA_2_ (Supplementary Figure S4B) confirms that only two of the four ATPase sites catalyze fast ATP hydrolysis and Pi release. Important questions that remain unresolved are: which two sites on UvrA_2_, proximal or distal, have burst activity, and what is the slow step that limits steady state turnover. These are addressed in the following sections.

**Figure 6.**
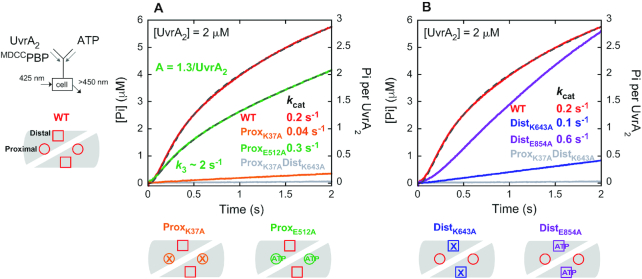
ATP binding to the proximal sites permits rapid ATP hydrolysis and Pi release by the distal sites. Pre-steady state Pi release kinetics were measured for UvrA_2_ proximal and distal site Walker A and B mutants as described for wild-type in Figure 5 (final: 2 μM UvrA_2_, 2 mM ATP and 15 μM ^MDCC^PBP); wild-type data and fit from Figure 5B are shown for comparison. (**A**) Proximal site mutants: ^K37A^UvrA_2_ has no burst and very low activity while ^E512A^UvrA_2_ shows a burst of Pi release followed by a slow linear phase at *k*_cat_ = 0.3 s^−1^ (slope/2 × [^E512A^UvrA_2_]); gray dashed line shows the fit to Scheme 1 (*k*_3_ = 2 ± 0.03 s^−1^, *k*_4_ = 42 ± 3 s^−1^, *k*_5_ = 0.6 ± 0.01 s^−1^, *n* = 1.3 ± 0.05 Pi/^E512A^UvrA_2_). (**B**) Distal site mutants: ^K643A^UvrA_2_ has no burst and very low activity while ^E854A^UvrA_2_ shows a lag followed by a fast linear phase at *k*_cat_ = 0.6 s^−1^ (slope/2 × [^E854A^UvrA_2_]). The double Walker A mutant ^K37A-K643A^UvrA_2_ has almost no detectable activity (gray trace).

The high ATP concentration required for a maximal burst either implicates the low affinity proximal sites in rapid ATP hydrolysis or implies that ATP binding to these sites is required for rapid ATP hydrolysis by the distal sites. These two possibilities were investigated by analyzing the UvrA_2_ ATPase mutants. The Walker A mutant ^K37A^UvrA_2_, in which ATP binding to proximal sites is disrupted while distal sites are intact, shows no burst and suffers near complete loss of activity (Figure 6A), consistent with an important role for the proximal sites in rapid ATP hydrolysis by UvrA_2_. However, the Walker B mutant ^E512A^UvrA_2_, in which proximal ATP binding remains intact but hydrolysis is disrupted, exhibits a burst of ATP hydrolysis and Pi release by the wild-type distal sites followed by linear steady state (*k*_cat_ = 0.3 s^−1^; linear slope/2 sites × [^E512A^UvrA_2_]), similar to wild-type UvrA_2_ (Figure 6A, Table 1). The data fit to the same mechanism in Scheme 1 yield *k*_3_ = 2 s^−1^, *k*_4_ = 42 s^−1^, *k*_5a_ = 0.6 s^−1^ and *n* = 1.3 Pi/^E512A^UvrA_2_, which differ at most 2-fold from wild-type parameters (Table 2). The corresponding distal site Walker A mutant ^K643A^UvrA_2_, in which distal ATP binding is disrupted while proximal sites are intact, shows no burst and has very low ATPase activity (Figure 6B). Moreover, the Walker B mutant ^E854A^UvrA_2_, in which distal ATP binding remains intact but hydrolysis is disrupted, exhibits a lag and no burst activity by the wild-type proximal sites. A faster *k*_cat_ = 0.6 s^−1^ (linear slope/2 sites × [^E512A^UvrA_2_]) suggests that the proximal ATPase is stimulated slightly when distal sites are ATP-bound (note: the proximal ATPase is inhibited when distal sites are ADP-bound; Supplementary Figure S5). Together these results confirm that the distal sites are responsible for the initial burst of ATP hydrolysis, and that ATP binding to the proximal sites is required for this activity. This finding was incorporated into Scheme 1B at step 3 (UvrA_2_•ATP_2P_•ADP_2D_•Pi_2D_). Scheme 1B also depicts the possibility that (i) only the two distal sites hydrolyze ATP per catalytic turnover or (ii) that the two proximal sites also hydrolyze ATP, except at a slow, turnover-limiting rate after burst hydrolysis by the distal sites (total four active sites per turnover). In best fit model, *k*_5a_ limits the steady state rate to 0.5 s^−1^ in case of two active sites, and *k*_5b_ limits it to 0.2 s^−1^ in case of four active sites (note that the measured *k*_cat_ of 0.2 s^−1^ for wild-type UvrA_2_ assumes four active sites per turnover; Table 1). Finally, the results also demonstrate that the proximal and distal sites are allosterically linked, since information about the nucleotide occupancy and ATPase activity of one site is communicated to the other site and influences its activity.

### Differential effects of native and lesion DNA on the proximal and distal site ATPase mechanisms

**Figure 7.**
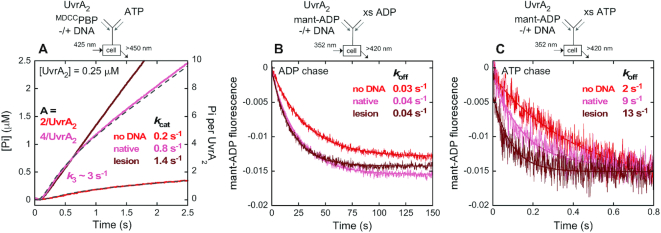
Native duplex DNA stimulates ATP hydrolysis by all four sites and subsequent ADP release limits turnover; fluorescein lesion DNA stimulates ADP release such that ATP hydrolysis or Pi release limits turnover. (**A**) Pre-steady state Pi release kinetics were measured by mixing UvrA_2_, DNA and ^MDCC^PBP with ATP (final: 0.25 μM UvrA_2_, +/- 1 μM DNA, 1 mM ATP and 15 μM ^MDCC^PBP). Without DNA, UvrA_2_ exhibits a burst of two Pi per dimer as in Figure 5. With native DNA, UvrA_2_ exhibits a higher burst amplitude, indicating rapid ATP hydrolysis by all four sites, followed by a linear phase at *k*_cat_ = 0.8 s^−1^ (slope/4 × [UvrA_2_]). Data at various UvrA_2_ concentrations in the presence of native DNA fit to Scheme 1 yield *k*_3_ = 2.4 ± 0.1 s^−1^, *k*_4_ = 30 ± 4 s^−1^, *k*_5_ = 1 ± 0.03 s^−1^, *n* = 5.4 ± 0.3 Pi/UvrA_2_; gray dashed line shows the fit. In contrast, lesion DNA stimulates UvrA_2_ ATPase activity but there is no burst, only a linear phase at *k*_cat_ = 1.4 s^−1^ (slope/4 × [UvrA_2_]). (**B** and **C**) Mant-ADP dissociation was measured by pre-incubating the nucleotide, UvrA_2_ and DNA, and mixing with excess unlabeled ADP or ATP chase (final: 0.1 μM UvrA_2_, 0.2 μM DNA, 10 μM mant-ADP and 2 mM ADP or ATP). The decrease in fluorescence over time was fit to a single exponential to determine *k*_off_. With ADP chase (**B**), native or lesion DNA has no significant effect on slow mant-ADP release from UvrA_2_ at *k*_off_ = 0.03–0.04 s^−1^. With ATP chase (**C**), however, mant-ADP is released >70-fold faster at *k*_off_ = 2.3 ± 0.1 s^−1^, and the rate is further accelerated by ∼4-fold to 9 s^−1^ and ∼6-fold to 13 s^−1^ with native and lesion DNA, respectively. These rate increases correlate with the DNA-induced increases in *k*_cat_ (A and Table 1).

A key question driving this study is how UvrA_2_ integrates its ATPase and DNA-binding activities to initiate NER. To address this question, we measured the ATPase kinetics of UvrA_2_ bound to two types of DNAs: a 50 bp undamaged (native) duplex and one with an identical sequence plus a centrally located fluorescein adduct (a fluorescein lesion is a good model substrate for bacterial NER) (20,61,62). Our measurements revealed that UvrA_2_ binds fluorescein lesion-containing DNA ∼4-fold tighter than native DNA (*K*_D_ = 12 nM versus 45 nM; Supplementary Figure S6), which is in line with earlier reports (24,63–68). Neither type of DNA appears to affect the nucleotide binding kinetics (Supplementary Figure S7; Table 1). However, both DNAs stimulate the steady state ATPase rate (linear phase in Figure 7A, Table 1) with *k*_cat_ = 0.8 s^−1^ and 1.4 s^−1^ for the native and the lesion DNA, respectively, compared with 0.2 s^−1^ for UvrA_2_ alone, indicating significant changes in the reaction mechanism (the same *k*_cat_ values were obtained from malachite green-based steady state experiments) (60); note: to avoid saturation of the ^MDCC^PBP reporter due to this high ATPase rate, pre-steady state measurements were performed at a lower UvrA_2_ concentration (0.25 μM) than in the absence of DNA (2 μM; Figure 5). In the presence of native DNA, UvrA_2_ again shows a short lag, a burst of ATP hydrolysis and Pi release, and then a linear steady state phase; however, the burst amplitude is higher than for protein alone (Figure 7A). The data were fit to the mechanism shown in Scheme 1, and in this case allowing the number of ATPase sites to float during fitting yields a burst amplitude of ∼5 ATP hydrolyzed per UvrA_2_ (*k*_3_ = 2.4 s^−1^, *k*_4_ = 30 s^−1^, *k*_5_ = 1 s^−1^, *n* = 5.4 ± 0.3 Pi/UvrA_2_). We interpret this result to mean that all four sites in the UvrA_2_–native DNA complex hydrolyze ATP rapidly (versus only the two distal sites in UvrA_2_ alone); the overestimate of five sites instead of four may arise from fitting error given the relatively small difference between the burst and linear rates. Also, while the step following Pi release is accelerated by native DNA (step 5), it remains slow enough to limit turnover. Figure 7A also shows that in the presence of fluorescein lesion DNA, UvrA_2_ ATPase kinetics change again, showing no burst activity, just a fast linear phase following a short lag. This result means that in the UvrA_2_–lesion complex, the slow step after ATP hydrolysis and Pi release has been accelerated and does not limit the turnover rate; instead, a step before or at ATP hydrolysis has become rate limiting.

Additional information on these rate-limiting step(s) is needed in order to understand what changes are induced by DNA in the UvrA_2_ ATPase mechanism. In many ATPases, catalytic turnover is associated with and limited by ADP release, and we hypothesized this might be the case for UvrA_2_ as well (also note that ADP co-purifies with UvrA_2_ and multiple ADP-bound UvrA_2_ structures have been solved, indicating high affinity). We expected ADP release rates to increase by about 4- and 7-fold in the presence of native and lesion DNA, respectively, corresponding to the increases in ATPase turnover rate noted above (*k*_cat_ = 0.2 s^−1^, 0.8 s^−1^ and 1.4 s^−1^ for UvrA_2_ alone, and with the native and lesion DNAs, respectively; Figure 7A, Table 1). However, as shown in Figure 7B, the rate of mant-ADP dissociation from distal sites on DNA-bound UvrA_2_ remains unchanged at 0.03 s^−1^, as observed in the absence of DNA (Figure 2C). Note that in these experiments we used excess unlabeled ADP as a passive chase to prevent mant-ADP rebinding to UvrA_2_ after dissociation. But, since the proximal and distal sites exhibit asymmetric nucleotide occupancy and allosteric communication, we also tested ATP as chase, wondering if ATP binding by the proximal sites might affect the mant-ADP bound at distal sites. Indeed, when excess unlabeled ATP is added to the UvrA_2_–mant-ADP complex in the absence of DNA, we observe ∼70-fold faster release of mant-ADP at 2 s^−1^ (Figure 7C). The presence of native and lesion DNA further stimulates mant-ADP release by 4- and 6-fold to 9 and 13 s^−1^, respectively (Figure 7C). While the absolute release rates of the mant-ADP analog are faster than the ATPase *k*_cat_ values (Table 1), the match between the relative increases in both rates induced by DNA strongly indicates that native and lesion DNA binding to UvrA_2_ alters the rate of ADP release following ATP hydrolysis and Pi release.

To summarize the results thus far with wild-type UvrA_2_: (i) ATP-bound proximal sites trigger ADP release from distal sites; (ii) in the absence of DNA, ADP release limits catalytic turnover following ATP hydrolysis by distal sites; (iii) when UvrA_2_ binds native DNA, ADP release from distal sites is accelerated following hydrolysis by all four sites, but is still slow enough to limit the turnover rate and finally, (iv) when UvrA_2_ binds a lesion, further acceleration of ADP release means an earlier step in the reaction (prior to/at ATP hydrolysis) becomes rate limiting instead of ADP release. In order to examine these findings in more detail and determine the effects of DNA on each ATPase site, we also analyzed the Walker A and B mutants in the presence of DNA as described below.

**Figure 8.**
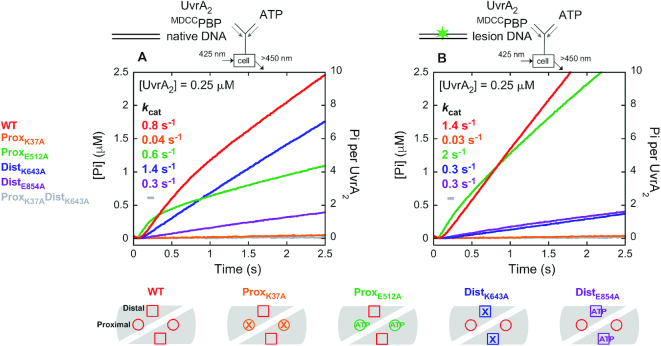
Native and fluorescein lesion DNAs have different effects on proximal and distal site ATPase mechanisms. Pre-steady state Pi release kinetics were measured for DNA-bound UvrA_2_ Walker A and B mutants as described for wild-type in Figure 7 (final: 0.25 μM UvrA_2_, 1 μM DNA, 1 mM ATP and 15 μM ^MDCC^PBP). (**A**) With the native duplex, ^K37A^UvrA_2_ shows no burst and has little activity, while ^E512A^UvrA_2_ exhibits a burst of ATP hydrolysis and Pi release followed by a slow linear phase at *k*_cat_ = 0.6 s^−1^ (slope/2 × [^E512A^UvrA_2_]), as in the absence of DNA (Figure 6A, green trace). ^K643A^UvrA_2_ and ^E854A^UvrA_2_ do not exhibit burst activity, and the linear rates differ than in the absence of DNA at *k*_cat_ = 1.4 s^−1^ and 0.3 s^−1^, respectively. (**B**) With lesion DNA, ^K37A^UvrA_2_ shows no burst and has little activity, while ^E512A^UvrA_2_ exhibits a slight burst and faster *k*_cat_ = 2 s^−1^ (slope/2 × [^E512A^UvrA_2_]). Both ^K643A^UvrA_2_ and ^E854A^UvrA_2_ show no burst and have low activity at *k*_cat_ = 0.3 s^−1^. The double Walker A mutant ^K37A-K643A^UvrA_2_ has little detectable activity (gray trace).

**Figure 9.**
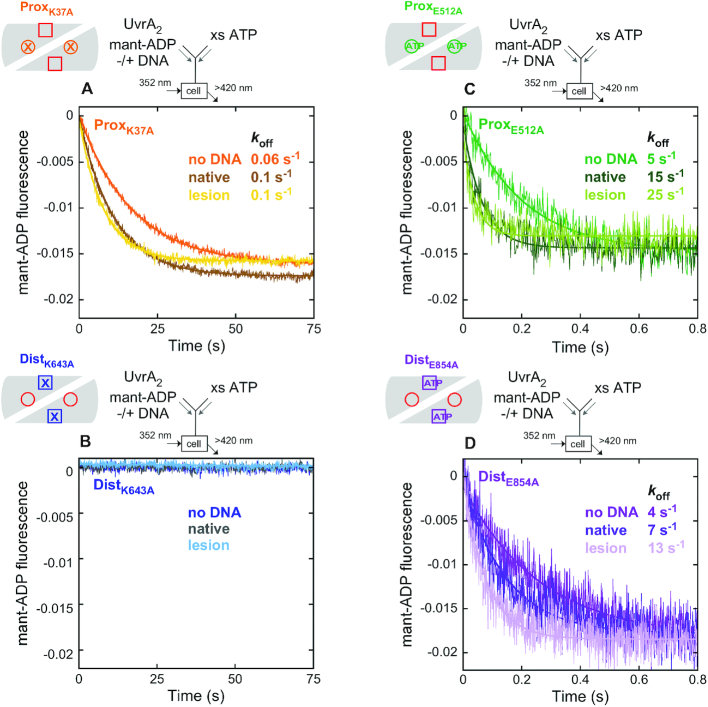
DNA stimulates ADP release from the distal site, only on ATP binding to the proximal site. Mant-ADP dissociation with ATP chase was measured for UvrA_2_ proximal and distal site Walker A and B mutants as described for wild-type in Figure 7C (final: 0.1 μM UvrA_2_, 0.2 μM DNA, 10 μM mant-ADP and 2 mM ATP). (**A**) ^K37A^UvrA_2_ exhibits slow mant-ADP release with or without DNA as it cannot bind the ATP needed to stimulate ADP release from distal sites. (**B**) ^K643A^UvrA_2_ exhibits no signal since proximal sites do not bind mant-ADP at the low concentration tested (Figure 3). (**C**) ^E512A^UvrA_2_ and (**D**) ^E854A^UvrA_2_ exhibit proximal site ATP and DNA binding-induced stimulation of mant-ADP release from distal sites as observed with wild-type UvrA_2_ (Figure 7C).

Figure 8A shows results from pre-steady state ATPase experiments with wild-type and mutant UvrA_2_ in the presence of native DNA. The Walker A mutant ^K37A^UvrA_2_, in which proximal ATP binding is disrupted while distal sites are intact, suffers near complete loss of activity, as seen in the absence of DNA (Figure 6A). The Walker B mutant ^E512A^UvrA_2_, in which proximal ATP binding is preserved but hydrolysis is disrupted, exhibits a burst of ATP hydrolysis and Pi release by the wild-type distal sites, followed by the linear steady state (*k*_cat_ = 0.6 s^−1^), again as seen in the absence of DNA (Figure 6A). These results show that in the UvrA_2_–native DNA complex, ATP binding to the weak proximal sites remains necessary for ATP hydrolysis by the distal sites, and that native DNA does not fundamentally alter the distal site ATPase mechanism, except to speed up the *k*_cat_ to some extent (likely by accelerating ADP release; Figure 9). The corresponding distal site mutants, ^K643A^UvrA_2_ and ^E854A^UvrA_2_, in which proximal sites are intact, do not exhibit any burst activity with native DNA, just as in the absence of DNA but, notably, the Walker A mutant ^K643A^UvrA_2_, in which distal ATP binding is disrupted, has a >10-fold faster ATPase rate (1.4 s^−1^ with DNA versus 0.1 s^−1^ without DNA; Figures 8A and 6B, respectively). This result shows that native DNA stimulates ATP hydrolysis and ADP release by the proximal sites on UvrA2 (when the distal sites are empty).

Next, Figure 8B shows results from experiments in the presence of fluorescein lesion-containing DNA. Again, the proximal site Walker A mutant ^K37A^UvrA_2_ shows near complete loss of activity, affirming that ATP binding to proximal sites is important for ATP hydrolysis by distal sites in the UvrA_2_–lesion DNA complex as well. The proximal site Walker B mutant ^E512A^UvrA_2_ exhibits a fast ATPase rate similar to wild-type UvrA_2_ (*k*_cat_ = 2 s^−1^), indicating that lesion DNA stimulates ADP release from distal sites and step(s) prior to ATP hydrolysis and Pi release become rate-limiting for catalytic turnover. In contrast, both distal site Walker A and B mutants, ^K643A^UvrA_2_ and ^E854A^UvrA_2_, which have intact proximal sites, exhibit low ATPase rates (*k*_cat_ = 0.3 s^−1^), indicating that unlike native DNA, a lesion suppresses proximal site ATPase activity.

Complementary experiments measuring mant-ADP release from UvrA_2_ in the presence of DNA confirm that ATP binding by proximal sites triggers ADP release from distal sites, and both native and lesion DNA further accelerate ADP release (Figure 9). Specifically, the data show that ^K37A^UvrA_2_ exhibits the slow, basal rate of mant-ADP release from the intact distal sites under all conditions, since the mutant proximal sites cannot bind ATP; DNA-bound ^E512A^UvrA_2_ exhibits faster mant-ADP release from the intact distal sites, since the mutant proximal sites can bind ATP; ^K643A^UvrA_2_ doesn’t show any change in signal, since the mutant distal sites cannot bind mant-ADP and the intact proximal sites do not bind mant-ADP stably at low concentrations; and finally, DNA-bound ^E854A^UvrA_2_ exhibits faster mant-ADP release from the mutant distal sites, since the intact proximal sites can bind ATP. The results from all the nucleotide binding and ATPase experiments with wild-type and mutant UvrA_2_ are summarized and interpreted in the context of available structural and biochemical data in the next section.

## DISCUSSION

**Figure 10.**
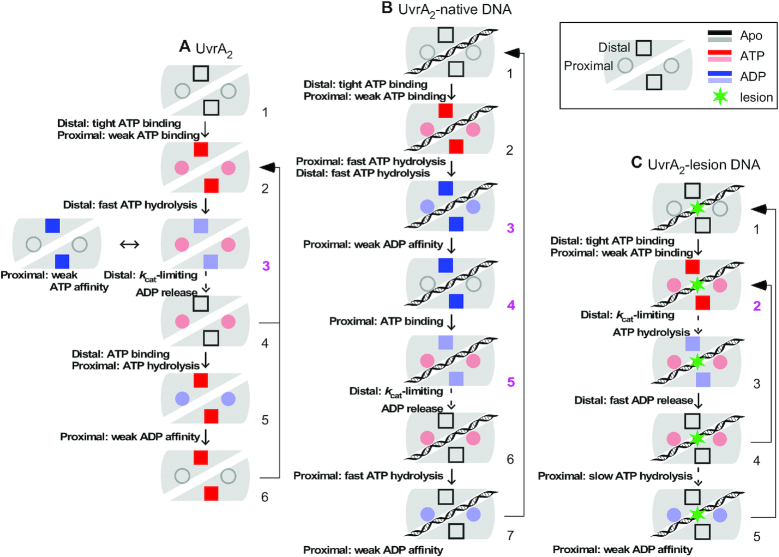
A summary of the differential effects of DNA on the UvrA_2_ ATPase mechanism. (**A**) Absent DNA, all ATPase sites on UvrA_2_, distal (D, tight binding = dark color) and proximal (P, weak binding = light color), binds ATP and only distal sites hydrolyze ATP and release phosphate rapidly. Rate limiting proximal ATP-induced ADP release from distal sites leads to accumulation of a P_2ATP_-D_2ADP_ species in steady state (A3). The proximal sites also hydrolyze ATP, but at a relatively slow rate especially when distal sites are empty, and perhaps not in every turnover. (**B**) When UvrA_2_ is bound to native DNA, both distal and proximal sites hydrolyze ATP and release Pi rapidly, resulting in a distinct, fully ADP-bound species, P_2ADP_-D_2ADP_ (B3); weak proximal affinity for ADP can lead to P_empty_-D_2ADP_ (B4); ADP release from distal sites remains rate limiting. (**C**) When UvrA_2_ is bound to a lesion, distal sites hydrolyze ATP and release both Pi and ADP rapidly; however, ATP hydrolysis by proximal sites is suppressed, thus these sites remain ATP-bound in the reaction through P_2ATP_-D_2ATP_, P_2ATP_-D_2ADP_, P_2ATP_-D_empty_ species (C2-C4), with P_2ATP_-D_2ATP_ (C2) having the longest lifetime. Drawing key is shown top right; pink numbers highlight UvrA_2_ species that may affect differential interactions with DNA.

The critical question of how UvrA_2_ ATPase activity is mechanochemically coupled to the search for lesions and initiation of DNA repair has been tackled previously (reviewed in (1)). However, UvrA_2_ presents a complex case as an ABC-type ATPase with two distinct active sites per monomer, and the workings of the two pairs of sites on the dimer are not resolved. The goal of this study was to determine, for the first time, the transient kinetics of ATP binding, hydrolysis and product release catalyzed by each site in three physiological contexts—free of DNA, and bound to native or lesion-containing DNA. Our results provide new information about the ATPase mechanism of each site, and how the sites are influenced by each other and by DNA. Based on these findings, and information from prior biochemical and structural studies, we propose a model of how each ATPase site contributes to UvrA_2_ function in NER (Figure 10).

### UvrA_2_ ATPase mechanism in the absence of DNA

We determined that the two distal sites on UvrA_2_ bind nucleotides tightly and hydrolyze ATP rapidly, whereas the two proximal sites bind nucleotides weakly and hydrolyze ATP slowly. This striking asymmetry is evident in the high affinity nucleotide binding and the exclusive burst of ATP hydrolysis and Pi release by the distal sites in the absence of DNA; as described earlier, inspection of a high resolution UvrA_2_ structure offers a molecular explanation for the differential activities of the two sites. We also found evidence of allosteric communication between the sites, the most striking being that ATP binding by the proximal sites is necessary for ATP hydrolysis, ADP release and catalytic turnover by the distal sites (note: the reverse is not true, but nucleotide occupancy at distal sites does alter proximal site activity to some extent as well). Interactions across the dimer linking Walker A and B sub-structures have been proposed previously as a conduit for communication between proximal and distal sites (26). Figure 10A shows the ATPase mechanism for apo UvrA_2_, wherein high affinity distal sites remain ATP-bound until ATP binding to the >300-fold lower affinity proximal sites (A2) triggers fast hydrolysis and Pi release (A3) followed by slower ADP release (A4), which limits catalytic turnover (this step also requires ATP-bound proximal sites). Meanwhile proximal sites hydrolyze ATP at a slower rate and release both products rapidly (A5, A6). It is unclear if UvrA_2_ has an obligate sequential ATPase mechanism, i.e. if proximal sites must hydrolyze ATP after each turnover of the distal sites, or if they hydrolyze ATP stochastically at a low frequency while the distal sites continue cycling (two pathways lead back to A2 in Figure 10A). In either case, P_2ATP_-D_2ADP_ (A3) accumulates in the reaction during steady state. Note that the low nucleotide affinity proximal sites may empty often, resulting in transient P_empty_-D_2ADP_ species, but at high ATP concentration the equilibrium should favor P_2ATP_-D_2ADP_.

UvrA_2_ ATPase activity has been linked to significant changes in its conformation and dynamics, as detailed below. UvrA_2_ structures from different organisms have been determined in nucleotide-free, P_2ADP_-D_2ADP_ or P_empty_-D_2ADP_ state, with most in open conformation (including the *T. maritima* UvrA_2_ structure reported here; Supplementary Figure S3A) with a shallow and wide DNA binding surface (23–26), and one structure (in complex with UvrB) in closed conformation with a deep and narrow DNA binding surface (15). Open UvrA_2_ can accommodate both native and damaged DNA, whereas the closed form appears to preclude binding of DNA distorted by damage (15). One important feature of ABC ATPase sites is the conserved signature domain (Figure 1B and C), which changes conformation in concert with ATP binding, hydrolysis and product release, coupling these events to changes in protein structure and interactions (69–72). In UvrA_2_, the proximal site signature domain II is notable as it presents cationic residues on the DNA-binding surface in both open and closed UvrA_2_ forms, and contributes to the UvrA_2_–UvrB binding interface as well (15,23). Nucleotide-bound/free UvrA_2_ structures reveal signature domain II in different conformations, suggesting it could affect ATPase-driven changes in UvrA_2_ interactions with DNA and UvrB. Such dynamism has not been reported for distal site signature domain I. A C-terminal zinc-binding hairpin within the proximal site signature domain II also adopts different conformations and is implicated in ATPase-modulated interactions of UvrA_2_ with damaged DNA (the UvrA_2_ structure reported here shows the hairpin in a new, intermediate position; Supplementary Figure S3B) (7,42). These movements depend more on distal site ATPase activity, illustrating an asymmetric allosteric effect of the distal site reaction on proximal site structure (42). Prior studies also indicate that UvrA_2_ dimerizes more effectively in the presence of ATP than non-hydrolyzable ATPγS or ADP (39), and that an ATPase-active distal site favors dimerization while the proximal site may be empty (33). UvrA_2_ also binds more specifically to lesions in the presence of ATP than ATPγS, ADP or no nucleotide (33,39). All of these results indicate the need for a mixed ATP/ADP/nucleotide-free UvrA_2_ species to initiate NER, and provide functional validation for our finding that the protein cycles through rapid ATP hydrolysis by the distal sites and produces a significant fraction of mixed P_2ATP_-D_2ADP_ (or P_empty_-D_2ADP_) dimer in steady state, ready to interact with DNA and locate a lesion either via 3D or combined 3D and localized 1D diffusion (5,6).

### UvrA_2_ ATPase mechanism in the presence of native DNA

Interaction between UvrA_2_ and native DNA has an asymmetric allosteric effect on the ATPase mechanism, with proximal sites undergoing significant change and distal sites less so. The proximal sites still bind nucleotide weakly, but ATP hydrolysis is accelerated when distal sites can hydrolyze ATP or are empty. Meanwhile, the distal sites still bind nucleotide tightly and require ATP binding by proximal sites for rapid ATP hydrolysis and Pi release. DNA stimulates ADP release from distal sites resulting in faster catalytic turnover, but this step remains rate-limiting. Thus, DNA-bound UvrA_2_ has the ATPase mechanism outlined in Figure 10B, wherein the distal sites remain ATP-bound until proximal ATP binding (B2) triggers fast hydrolysis and Pi release by all four sites (B3). The weak proximal sites release ADP (B4), and subsequent ATP binding (B5) enables ADP release from distal sites (B6). This in turn promotes ATP hydrolysis by proximal sites (B7) and fast ADP release to continue the reaction cycle. Notably, due to DNA-induced changes in the mechanism, UvrA_2_ transitions more rapidly through the ATPase reaction and through species less likely to form in the absence of DNA, such as P_2ADP_-D_2ADP_ (B3) and P_empty_-D_2ADP_ (B4). Also, since distal ADP release is still rate-limiting, the P_2ATP_-D_2ADP_ species (B5) can accumulate in steady state as in the absence of DNA, except the turnover rate is faster.

UvrA_2_ structures determined in open form are in either nucleotide-free (23,24), fully ADP-bound (25,26) or, in one case, in P_empty_-D_2ADP_ state (15), suggesting that ATP hydrolysis by one or both ATPase sites favors this conformation (the fully ADP-bound structure reported here further supports this hypothesis; Supplementary Figure S3A). The closed form of UvrA_2_ has nucleotides in all four sites, but their identity is not resolved (15). As noted earlier, the structures indicate that open UvrA_2_ can bind both native and damaged DNA, whereas the closed form cannot accommodate DNA distorted by damage. It has been proposed that UvrA_2_ cycles repeatedly through open-closed forms to check the duplex for lesions as it cycles through the ATPase reaction (15). Past studies indicate that UvrA_2_ binds native DNA non-specifically in the absence or presence of ATP, ATPγS or ADP, but ATPγS favors and ADP impairs the interaction (33,34), and distal site ATPase activity in particular weakens it (34). Accordingly, we propose that changes in the ATPase mechanism on binding native DNA, i.e. ATP binding followed by hydrolysis by all four sites and accelerated ADP release, allow UvrA_2_ to transition faster through ATP (closed)- and ADP (open)-bound forms that have higher and lower affinity for the duplex, respectively, thereby enabling lesion search. Furthermore, the P_empty_-D_2ADP_ UvrA_2_ structure shows outward rotation of signature II domain away from ATP binding domain I, which disrupts some critical DNA contacts (15). We speculate that native DNA-induced stimulation of proximal ATP hydrolysis and ADP release promotes formation of this UvrA_2_ species, and the resulting transient dissociation from DNA enables the 3D/localized 1D diffusion search mechanism proposed recently based on single molecule imaging data (5).

### UvrA_2_ ATPase mechanism in the presence of damaged DNA

Encounter with a lesion in DNA has a different allosteric effect on UvrA_2_, with both sites undergoing significant, distinct changes in their ATPase mechanism. ATP hydrolysis is suppressed at the proximal sites, irrespective of nucleotide occupancy at distal sites. The distal sites still require proximal ATP binding to catalyze ATP hydrolysis and Pi release, but ADP release is accelerated and no longer limits the turnover rate. Thus, lesion-bound UvrA_2_ has the ATPase mechanism outlined in Figure 10C, wherein the distal sites remain ATP-bound until proximal ATP binding (C2) triggers fast ATP hydrolysis as well as Pi and ADP release by distal sites (C3, C4). The proximal sites are stabilized in an ATP-bound state with low catalytic activity (C4, C5), while distal sites can continue to turnover. Moreover, since steps prior to ATP hydrolysis and product release by the distal site are now rate limiting, distal nucleotide-free or ATP-bound states have longer lifetime than ADP-bound states, which favors UvrA_2_ species such as P_2ATP_-D_empty_ (C4) and P_2ATP_-D_2ATP_ (C2), and high ATP concentration favors P_2ATP_-D_2ATP_.

After finding a lesion, UvrA_2_ must orchestrate its hand-off to UvrB for lesion verification. As noted earlier, outward movement of signature II domain in the P_empty_-D_2ADP_ open UvrA_2_ form can disrupt interactions with DNA and UvrB, implying that ATP binding by proximal sites promotes these interactions (15). Consistent with this interpretation, the proximal Walker A mutant has low affinity for UvrB and is defective in loading UvrB on the lesion, whereas the distal Walker A mutant is less affected (15,33). We propose that changes in the UvrA_2_ ATPase mechanism on lesion binding, i.e. stabilization of the ATP-bound proximal site and ongoing catalytic turnover by the distal site allows UvrA_2_ to remain localized at the lesion and recruit UvrB (38). Subsequent ATP hydrolysis and ADP release by the proximal site would reset the signature II domain, promoting UvrA_2_ dissociation and UvrB access to the lesion. The only crystal structure of UvrA_2_ bound to DNA containing a pair of opposing fluorescein lesions is in open form (23), with all four ATPase sites empty and signature II domain in a distinct conformation compared with P_2ADP_-D_2ADP_ and P_empty_-D_2ADP_ structures (15,26). Based on the ATPase mechanism of lesion-bound UvrA_2_ described here, this structure could reflect an early state, before lesion specific interactions promote ATP-bound proximal sites and ATP hydrolysis by the distal sites for recruiting UvrB. Alternately, the structure could reflect a late state after ATP hydrolysis and ADP release, ready to dissociate from DNA and make room for UvrB at the lesion.

### A mechanochemical model of ATPase-driven UvrA_2_ actions during NER

The ATPase kinetic mechanism determined in this study offers a view of the mechanochemical coupling in UvrA_2_ as it searches for damage lesions in DNA and initiates NER. As seen in other well-studied ATPases, including ABC transporters (30), each step is closely linked to the next in both the mechanical and chemical cycles. For UvrA_2_, we posit that ATP binding increases affinity for DNA, and DNA binding in turn promotes UvrA_2_ closure around a native duplex, which triggers ATP hydrolysis, followed by opening of ADP-bound/nucleotide-free UvrA_2_ with weakened contacts and lower affinity for the duplex, until an ADP–ATP switch resets the cycle and the protein can continue scanning DNA. Lesion binding would block UvrA_2_ closure, which in turn would alter the ATPase mechanism and maintain UvrA_2_ in proximal ATP-bound form with higher affinity for DNA and UvrB, setting the stage for UvrB entry. Presumably, a subsequent event would trigger ATP hydrolysis and ADP release, resulting in lesion hand-off to UvrB and UvrA_2_ exit. While many of the transient events in this model have not been explicitly measured, the UvrA_2_ ATPase mechanism presented here will facilitate more specific interpretation of structural and kinetic data on its interactions with DNA and UvrB, and related conformational dynamics, to understand how this protein uses ATP to initiate NER.

## DATA AVAILABILITY

Atomic coordinates and structure factors for the reported crystal structure have been deposited with the Protein Data Bank under the accession number 6N9L.

## Supplementary Material

Supplementary Data
