# Supplementary material for: The ATPase mechanism of UvrA2 reveals the distinct roles of proximal and distal ATPase sites in nucleotide excision repair
Source: Nucleic Acids Res. 2019 Mar 20;47(8):4136–52. doi: 10.1093/nar/gkz180 (PMC6486640; doi:10.1093/nar/gkz180)
Supplement: Supplementary Data [file gkz180_supplemental_files.zip › Case_Rev2_SupplInfo.pdf]

The ATPase mechanism of $UvrA_2$ reveals the distinct roles of proximal and distal ATPase sites in nucleotide excision repair

Brandon C. Case[1], Silas Hartley[2,3], Memie Osuga[2,4], David Jeruzalmi[2,3,5] and Manju M. Hingorani[1]*

[1] Department of Molecular Biology and Biochemistry, Wesleyan University, Middletown, Connecticut 06459, USA.

[2] Department of Chemistry and Biochemistry, City College of New York of the City University of New York, New York, NY 10031, USA.

[3] Ph.D. Program in Biochemistry, The Graduate Center of the City University of New York, New York, NY 10016, USA.

[4] Hunter College High School, New York, NY 10128, USA.

[5] Ph.D. Programs in Chemistry and Biology, The Graduate Center of the City University of New York, New York, NY 10016, USA.

*To whom correspondence should be addressed:
Phone: (860) 685-2284; Email: mhingorani@wesleyan.edu

SUPPLEMENTARY METHODS

Analysis of nucleotide contaminants in purified *G. stearothermophilus* $UvrA_2$ protein

Samples (100 µl) containing 250 nM $UvrA_2$ were treated with 1 U proteinase K (6 hrs at 37 °C), heat denatured and cooled to 25 °C, and then incubated with 5 U pyruvate kinase and 1 µM phosphoenolpyruvate (Millipore Sigma) for 30 minutes at 37 °C to convert any ADP to ATP. The samples were then mixed in a 1:1 ratio with 25-fold diluted detection buffer provided in the assay kit (Millipore Sigma). The luminescence was measured on a scintillation counter immediately after addition of detection buffer. The signal was converted to ATP concentration using a calibration curve generated under the same conditions with an ADP solution.

DNA binding to *G. stearothermophilus* $UvrA_2$ protein

Damaged DNA containing a mid-sequence fluorescein lesion or undamaged DNA labeled at the 5′ end with 6-FAM (7.5 nM) was titrated with $UvrA_2$ (0 - 250 nM) in individual cuvettes, in buffer (20 mM Tris-HCl, pH 7.4, 0.15 M NaCl, 5% glycerol, 5 mM $MgCl_2$, 5 mM DTT) containing 2 mM ATP. After mixing the reagents and equilibrating for 1 minute at 40 °C, the samples were excited with vertically polarized light (λ_{EX} = 494 nm, λ_{EM} = 518 nm), and the change in fluorescence anisotropy was calculated from the emitted vertical (I_{VV}) and horizontal (I_{VH}) polarized fluorescence intensities ($I_{VV} - GI_{VH}/I_{VV} + 2GI_{VH}$; G is the grating correction factor). Average values from three independent experiments were plotted versus $UvrA_2$ concentration,

and the binding isotherms were fit to a quadratic equation for 1:1 binding to determine the dissociation constants (1).

Global fitting of UvrA$_2$ ATPase kinetic data by KinTek and FitSpace Explorer.

A minimal model was developed that best fit the data obtained from pre-steady state kinetic analysis of UvrA$_2$ ATPase activity. We used KinTek Explorer (2,3), which simultaneously fits multiple datasets based on numerical integration of rate equations describing the model (4). Pi release data from both the ATP (Figure 5) and UvrA$_2$ (Figure S4B) titrations were fit to the same model. The derived kinetic parameters were analyzed further by FitSpace Explorer (5) to determine if they were sufficiently constrained by the data.

The model (Scheme 1a) begins with ATP binding to UvrA$_2$ (step 1) at a bimolecular rate constant estimated at 5 x 10^4 M^{-1} s^{-1} (k_1) and dissociating at 0.05 s^{-1} (k_{-1}), based on mant-nucleotide binding and dissociation kinetics (Figure 2). These rate constants were linked to maintain a tight K_{D1} of 1 μM and allowed to float during data fitting by KinTek Explorer (note: the entire range of measured K_{D1} estimates for the high affinity site, from 0.23 - 5 μM, was tested during fitting and yielded the same results). Another ATP binding step was added (step 2), based on evidence that while two of the four sites on UvrA$_2$ bind ATP tightly (Figure 2), a high concentration of ATP is required for maximal burst ATPase activity (Figure 5), indicating a distinct weak ATP binding event in the reaction (note: ATP hydrolysis after the first binding step is negligible, as indicated by the 0.04 s^{-1} ATPase rate of ^{K37A}UvrA$_2$ mutant compared to 2.4 s^{-1} for wild type UvrA$_2$; Figure 6). For the second ATP binding step, K_{D2} (k_{-2}/k_2) was estimated at 350 μM and linked to K_{D1} during data fitting (Figure 5). Minimally, two additional steps were required before turnover for the model to fit the observed lag and burst phases in the data. These were designated as ATP hydrolysis (step 3; k_3) and Pi release (step 4; k_4), and the rate constants were allowed to float (note: an alternative model in which ATP hydrolysis and Pi release were consolidated into one step did not fit the data as well, especially the lag phase). Both steps were considered irreversible in the model since the reverse rates are unknown and expected to be very small. After the burst of Pi release, a slow step in the reaction (step 5; k_5) limits the steady state turnover rate. This step was designated as ADP product release from UvrA$_2$, based on observed correspondence between DNA-induced changes in both mant-ADP dissociation and steady state ATPase rates (Figure 7), and the rate constant was allowed to float. Inclusion of a reverse step in the model (k_{-5}; rebinding of ADP estimated at 1 x 10^5 M^{-1} s^{-1} from mant-ADP binding measurements) did not alter the results from data fitting. Since the number of ATP molecules hydrolyzed rapidly within a single catalytic turnover was unknown, a stoichiometric coefficient (n) was applied as scaling factor to the Pi concentration output and allowed to float during data fitting.

In addition to standard error analysis based on nonlinear regression, confidence contour analysis was performed to assess whether the data are sufficient to define well constrained parameters in the model, especially those lacking independent measurements at this time. FitSpace Explorer assesses the extent to which each rate constant can vary while the others float in search of the best fit, and the extent to which each pair of rate constants can co-vary and produce a good fit. The results are scored by the minimal attainable χ^2 value, and a 5% increase in χ^2 was used as the boundary to set confidence limits for each parameter. The best fit values for the unknown

rate constants are listed in Scheme 1b and Table 2, which also lists the standard errors from nonlinear regression, and the upper and lower limits from confidence contour analysis.

Scheme 1b shows additional details, including identification of the tight/fast (distal) and weak/slow (proximal) ATPase sites from analysis of $UvrA_2$ Walker A and B mutants. It also shows two options for step 5 in the best fit model: either rate-limiting ADP release from the two distal sites leads to catalytic turnover without ATP hydrolysis by the proximal sites (step 5a; k_{5a}), or rate-limiting ATP hydrolysis by the proximal sites and product release from all four sites leads to catalytic turnover (step(s) 5b; net rate k_{5b}). Thus, the steady state rate is limited by k_{5a} = 0.5 s^{-1} if two ATPase sites per $UvrA_2$ are active per turnover, or by k_{5b} = 0.2 s^{-1} if four ATPase sites per $UvrA_2$ are active per turnover. Values of the other rate constants remain the same in either case. Note that for analysis of $^{E512A}UvrA_2$ ATPase data, step 5a is the only option since the two proximal sites are not catalytically active. Also, for native DNA-bound $UvrA_2$, step 5 represents ADP release after rapid ATP hydrolysis and Pi release by all four sites.

Preparation and crystallization of *T. maritima* $UvrA_2$ protein

Cloning

The wild type *T. maritima* UvrA pET11a clone was a gift from Nora Goosen (Leiden University). An N-terminal His-tag was added using the QuikChange XL Site-Directed Mutagenesis Kit (Agilent Technologies) and the following primers: forward: 5′-GAA GGA GAT ATA CAT ATG CAT CAT CAC CAT CAC CAC AAC GAA ATC GTG GTG AAA GG-3′ and reverse: 5′-CCT TTC ACC ACG ATT TCG TTG TGG TGA TGG TGA TGA TGC ATA TGT ATA TCT CCT TC-3′.

A truncated construct of *T. maritima* UvrA was designed based on the previously studied *G. stearothermophilus* UvrA Δ118-419 construct, in which the UvrB binding domain and part of the signature I domain are removed (6). The equivalent construct, *T. maritima* UvrA Δ117-399, was determined by sequence comparison with *G. stearothermophilus UvrA* using Clustal Omega (7), and prepared using the QuikChange XL Site-Directed Mutagenesis Kit (Agilent Technologies) and the following primers: forward: 5′-GCT CTA CGC GAG GAT AGG AAA AAA GAT AAA CGG TTT GAA CAT ACA CG-3′ and reverse: 5′-CGT GTA TGT TCA AAC CGT TTA TCT TTT TTC CTA TCC TCG CGT AGA GC-3′. All primers were purchased from Integrated DNA Technologies, Inc. and the clones were sequenced by Genewiz, Inc.

Purification

T. maritima UvrA Δ117-399 (pET11a-NHis-UvrA) was expressed in *E. coli* Rosetta (DE3) pLysS cells. Cells were grown in Super Broth (homemade) at 37 °C with O_2 supplied at 0.5 L/minute to an OD_{600} of 3.94, and induced overnight at 16 °C with 1 mM IPTG. Cell pellets were resuspended (at 5 mL/g) and lysed as described previously, except with 0.5 M NaCl added prior to sonication (8). The soluble fraction was incubated with Ni-NTA agarose beads (Qiagen) for 45 minutes, washed with buffer (40 mM sodium phosphate, pH 7.5, 0.5 M NaCl and 5% glycerol) containing up to 40 mM imidazole and eluted with 250 mM imidazole. Ammonium phosphate was added to the eluate to 1.5 M, followed by chromatography on a Macro-Prep Methyl HIC column (Bio-Rad Laboratories Inc.). Protein was eluted with an 8-column volume gradient of 1.5 M to 0 M ammonium sulfate in buffer (40 mM sodium phosphate, pH 7.5, 5%

glycerol, 0.5 mM EDTA, 1 M NaCl and 1 mM DTT). Fractions containing UvrA Δ117-399 were pooled, concentrated, and further purified by size-exclusion chromatography on a Superdex 200 column (GE healthcare Life Sciences) in buffer (20 mM HEPES-NaOH, pH 7.0, 1 M NaCl, 5% glycerol, 50 mM Arg, 50 mM Glu and 1 mM DTT). The protein was flash frozen in liquid nitrogen and stored at -80 °C.

Crystallization

Crystallization and analysis were performed using instrumentation available at the Structural Biology Initiative at the Advanced Science Research Center (The Graduate Center: City University of New York). Crystals of purified *T. maritima* UvrA Δ117-399 (14.83 mg/mL) were prepared using the sitting drop vapor diffusion method by mixing 0.1, 0.2, or 0.4 µL of the protein solution (20 mM HEPES-NaOH, pH 7.0, 1 M NaCl, 50 mM Arg, 50 mM Glu, 1 mM DTT, 1 mM ATP, 2 mM $MgCl_2$) and 0.2 µL of the reservoir solution from a ComPAS screen (Qiagen; 100 mM HEPES-NaOH, pH 7.5 and 22 w/v PEG 3350). Crystals were flash frozen in liquid nitrogen after a short wash (<5 minutes) with a cryoprotecting solution (20% glycerol, 20 mM HEPES-NaOH, pH 7.0, 1 M NaCl, 50 mM Arg, 50 mM Glu, 1 mM DTT, 1 mM ATP, 24 w/v PEG 3350, 100 mM HEPES-NaOH, pH 7.5) within 2 weeks.

Data collection and structural analysis

Diffraction data was collected at the Brookhaven National Laboratory on the Bl-12 line by Vivian Stojanoff, PhD (Physicist, National Synchrotron Light Source II) using a wavelength of 0.97946 Å. Diffraction data was reduced to a C121 space group, with the following cell parameters: a = 143.134 Å, b = 81.379 Å and c = 90.416 Å, α = 90°, β = 125.3°, γ = 90° using HKL2000 (9). Matthews analysis revealed one protein unit in the asymmetric unit (V_m = 3.06 A^3/Da). The structure was determined to a resolution of 2.0 Å using the following features of the Phenix software suite: molecular replacement, using the four UvrA domains in PDB 3UX8 as search models, and crystallographic refinement (Phenix.refine) (10-12). Nucleotides were built in the electron density by hand using the molecular visualization program COOT (13). The final model of *T. maritima* UvrA Δ117-399 includes residues 1-60, 69-117 and 399-916. The model has an *R*-factor of 17.78% and an R-free of 20.79%.

Structural analysis of the ATPase sites was performed in PyMol (The PyMOL Molecular Graphics System, Version 2.0 Schrödinger, LLC.) and Maestro 11.7 ligand interaction protocol with a 4 Å cutoff (Schrödinger Release 2018-3: Maestro, Schrödinger, LLC, New York, NY, 2018). A search for PDBs with similar ABC signature motifs was conducted using ScanProsite (14). The β-hairpin loop was compared using DynDom (15).

SUPPLEMENTARY FIGURE LEGENDS

Figure S1. Testing for nucleotide contamination in purified $UvrA_2$ protein. Histogram showing ATP detected in $UvrA_2$ samples by a luciferase-based ATP detection assay (Millipore Sigma) coupled with pyruvate kinase (PK) and phosphoenolpyruvate (PEP) to convert any ADP present into ATP. (1) buffer control; (2) ADP (10 pmoles) with PK and PEP; (3, 5) $UvrA_2$ (25 pmoles) purified without heat treatment, with and without PK and PEP, respectively; (4, 6) $UvrA_2$ (25 pmoles) purified with a 55 ºC heat treatment step, with and without PK and PEP, respectively.

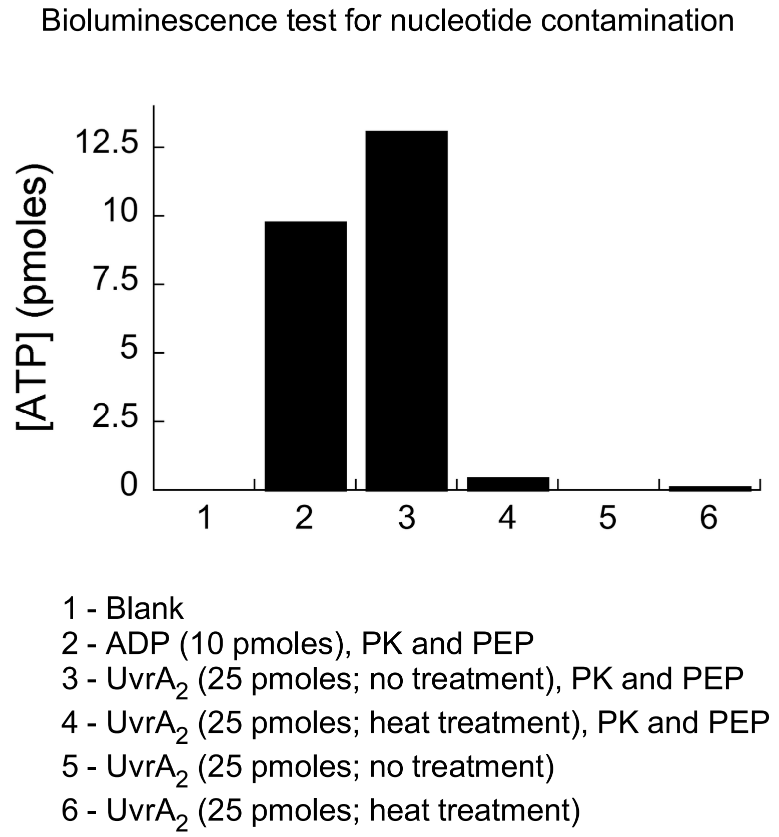

Figure S2. Kinetics of nucleotide binding to wild type (WT) and mutant $UvrA_2$. **(A)** $UvrA_2$ structure showing proximal and distal site Walker A (K37, K643) and B (E512, E854) residues, respectively (PDB code: 2R6F). Nucleotide binding kinetics were measured by increase in mant fluorescence over time on mixing $UvrA_2$ with varying concentrations of **(B)** mant-ATP or **(C)** mant-ADP on a stopped flow (final concentrations: 0.1 μM $UvrA_2$ and 2.5, 5 and 10 μM mant-ATP or mant-ADP); traces shown for WT $UvrA_2$. The time traces fit to a single exponential yield rates that depend linearly on **(D)** mant-ATP and **(E)** mant-ADP concentration and provide comparable bimolecular binding rate constants for WT and mutant $UvrA_2$, except $^{K643A}UvrA_2$ which does not exhibit any binding at the highest concentrations tested (see Figures 2, 3 and Table 1). **(F)** Nucleotide binding stoichiometry was measured by FRET on titrating $UvrA_2$ or $^{K643A}UvrA_2$ (3 μM) with increasing concentrations of mant-ADP (0 - 10 μM; see Figure 2). With wild type protein, the increase in FRET yields a binding isotherm with an inflection point of 6 μM (2 mant-ADP per $UvrA_2$ dimer), whereas with distal site Walker A mutant, a small, linear increase in FRET over the same concentration range indicates weak/non-specific mant-ADP binding.

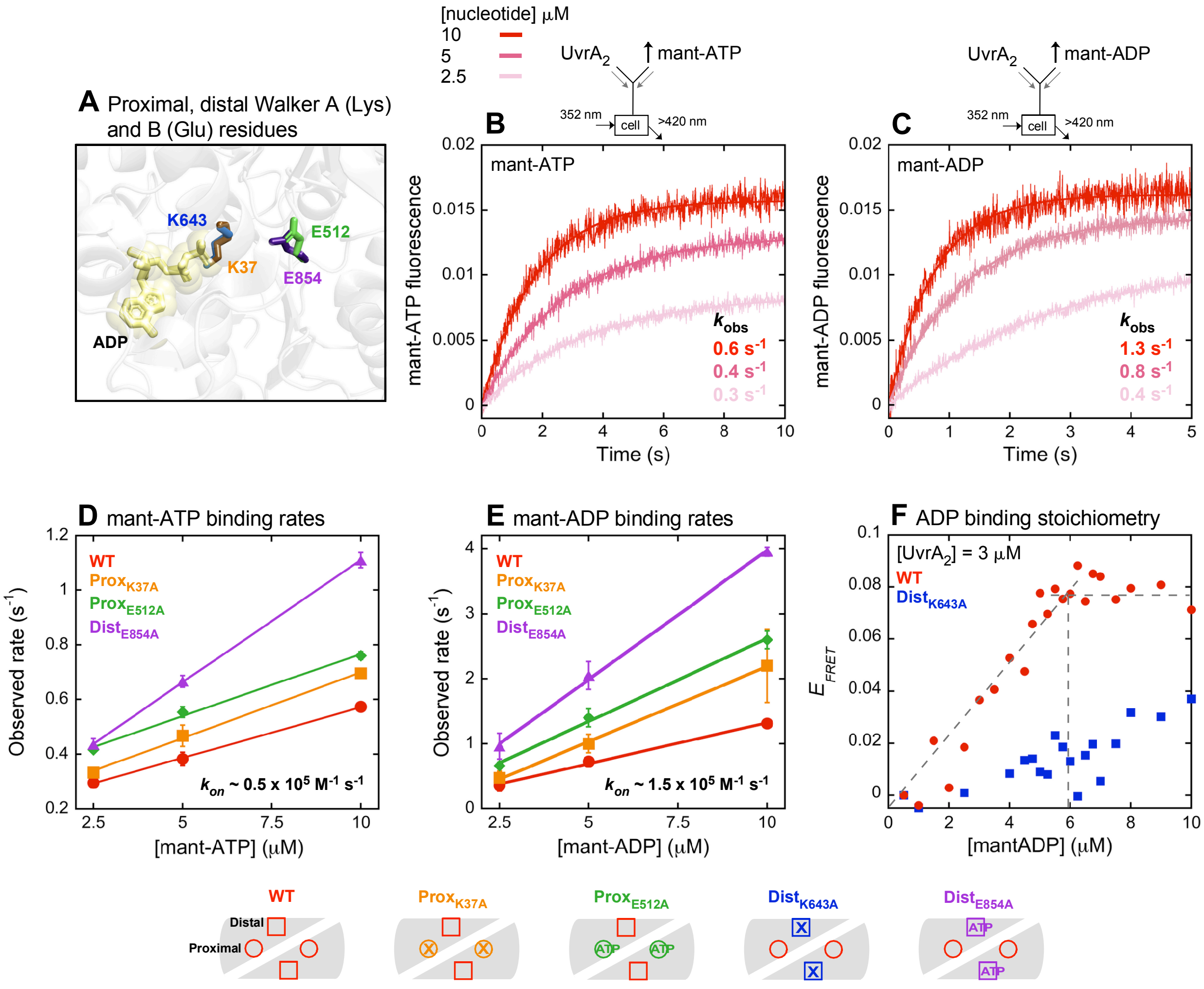

Figure S3. (A) The structure of *T. maritima* Δ117-399 $UvrA_2$. The $UvrA_2$ dimer, component domains, ADP, and path of DNA are depicted using the same coloring scheme as in Figure 1B. **(B)** Conformational heterogeneity in the β-hairpin of signature domain II as seen in various crystal structures of $UvrA_2$. The signature-II domains of each $UvrA_2$ ortholog are shown in color: *G. stearothermophilus*, purple (PDB entry = 2R6F) (16), *T. maritima* Δ117-399, pink (PDB entry = 6N9L), and *T. maritima* complex with DNA in green (PDB entry = 3PIH) (8). Movements of the signature-II domains are demonstrated in both top- and side-views of $UvrA_2$ (highlighted by the different positions of the Zinc atoms) and show the β-hairpin of *T. maritima* $UvrA_2$ Δ117-399 occupying an intermediate position (movement and rotation from DynDom are reported). Other segments of $UvrA_2$ are shown as a faded white surface.

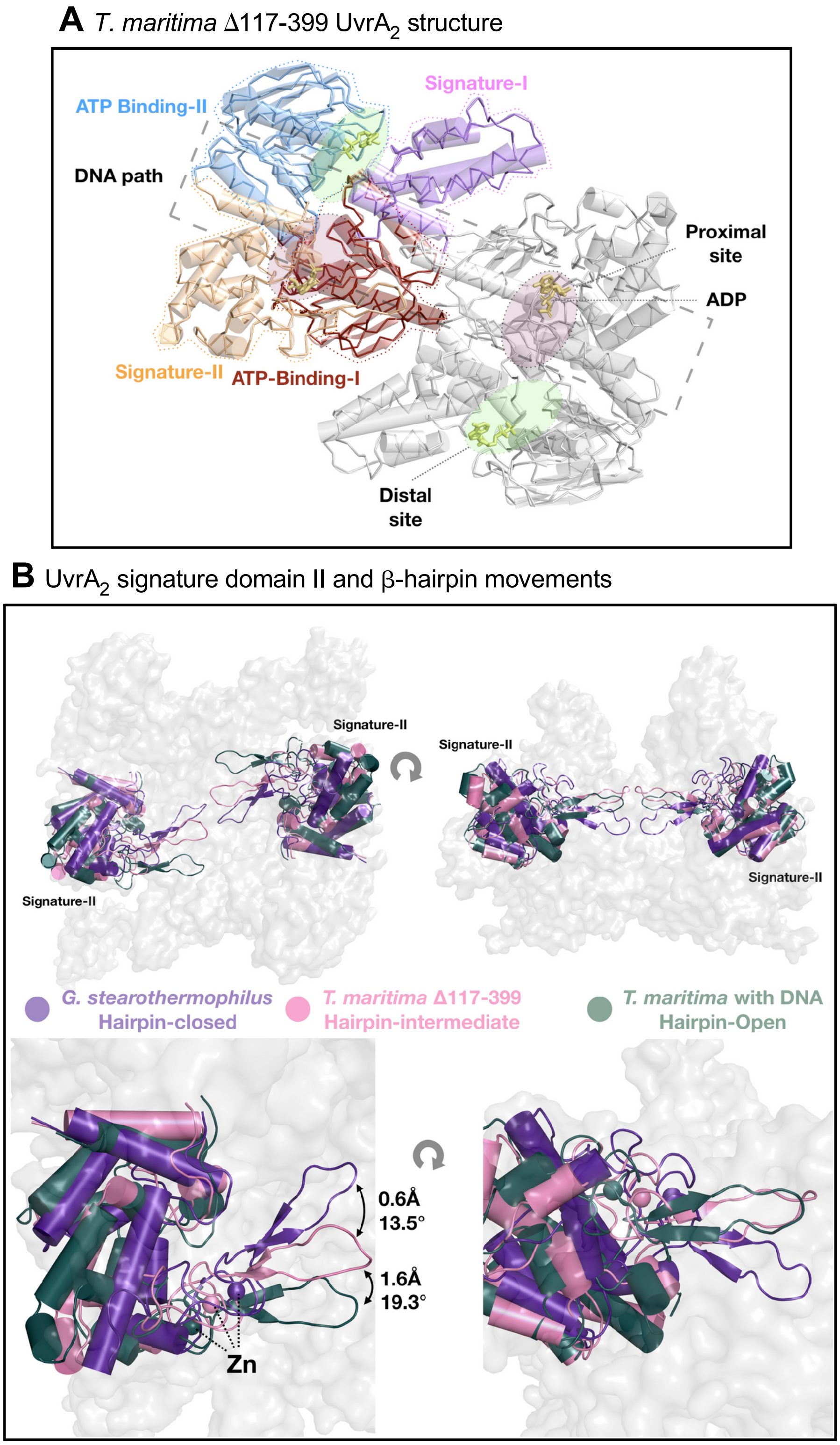

Figure S4. Analysis of $UvrA_2$ ATPase kinetics. **(A)** A calibration plot of ^{MDCC}PBP fluorescence versus Pi concentration used to determine the amount of Pi produced in the ATPase experiments. **(B)** Pre-steady state kinetics of ATP hydrolysis and Pi release were measured by mixing increasing concentrations of $UvrA_2$ and ^{MDCC}PBP reporter with ATP on a stopped-flow (final concentrations: 0.125 - 2 μM $UvrA_2$, 1 mM ATP and 15 μM ^{MDCC}PBP). All traces – from the $UvrA_2$ titration shown here and the ATP titration (10 μM - 2 mM ATP) shown in Figure 5 – were fit to the kinetic mechanism in Scheme 1 using KinTek Explorer; Scheme 1 is repeated here for reference. The fits are shown as dashed lines in the graph and the burst of Pi/$UvrA_2$ at each $UvrA_2$ concentration is listed. **(C)** FitSpace Explorer was used to derive confidence contours for the four unknown rate constants obtained from the fit, k_1 (linked with k_{-1}, k_2, k_{-2}), k_3, k_4 and k_5, which determined the extent to which each rate can vary while producing a good fit (5). The plots represent the χ^2 variation for each pair of rate constants. The red zone shows that each pair is well constrained to a local minimum. A 5% increase in χ^2 values, depicted by the yellow zone, was used to determine the upper and lower confidence limits on each rate shown in Table 2. Interestingly, k_3 and k_5 appear linearly correlated, which suggests that a constant factor in the mechanism governs ADP formation (k_3) release (k_5) at the distal site. **(D)** Comparison of three fits to the Pi release data (2 μM $UvrA_2$, 1 mM ATP): (i) a double exponential+linear function, which yielded initial estimates of rate constants, (ii) Scheme 1 (as in panel **B**), and (iii) Scheme 1-alt, in which steps 3 and 4 (ATP hydrolysis and Pi release) were consolidated into one step. As observed on the expanded 1 second time scale, Scheme 1-alt does not yield a good fit, especially for the lag phase, confirming the need for two intermediate steps between ATP binding and steady state turnover in the minimal model (Scheme 1).

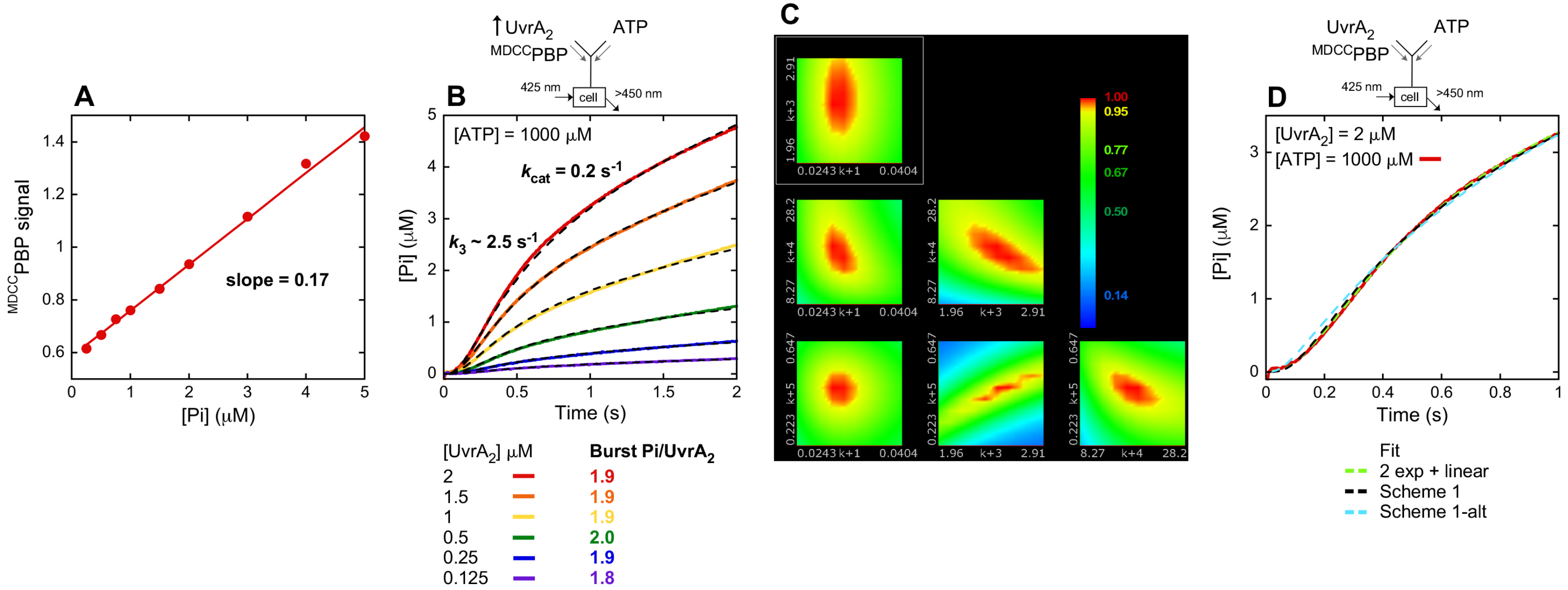
A
B
C
D
MDCCPBP signal
slope = 0.17
[Pi] (μM)
↑UvrA2
MDCCPBP
ATP
425 nm
cell
>450 nm
[ATP] = 1000 μM
kcat = 0.2 s-1
k3 ~ 2.5 s-1
[Pi] (μM)
Time (s)
[UvrA2] μM
Burst Pi/UvrA2
2 1.9
1.5 1.9
1 1.9
0.5 2.0
0.25 1.9
0.125 1.8
1.00
0.95
0.77
0.67
0.50
0.14
k+1
k+3
k+4
k+5
UvrA2
[UvrA2] = 2 μM
[ATP] = 1000 μM
Fit
2 exp + linear
Scheme 1
Scheme 1-alt

Scheme 1

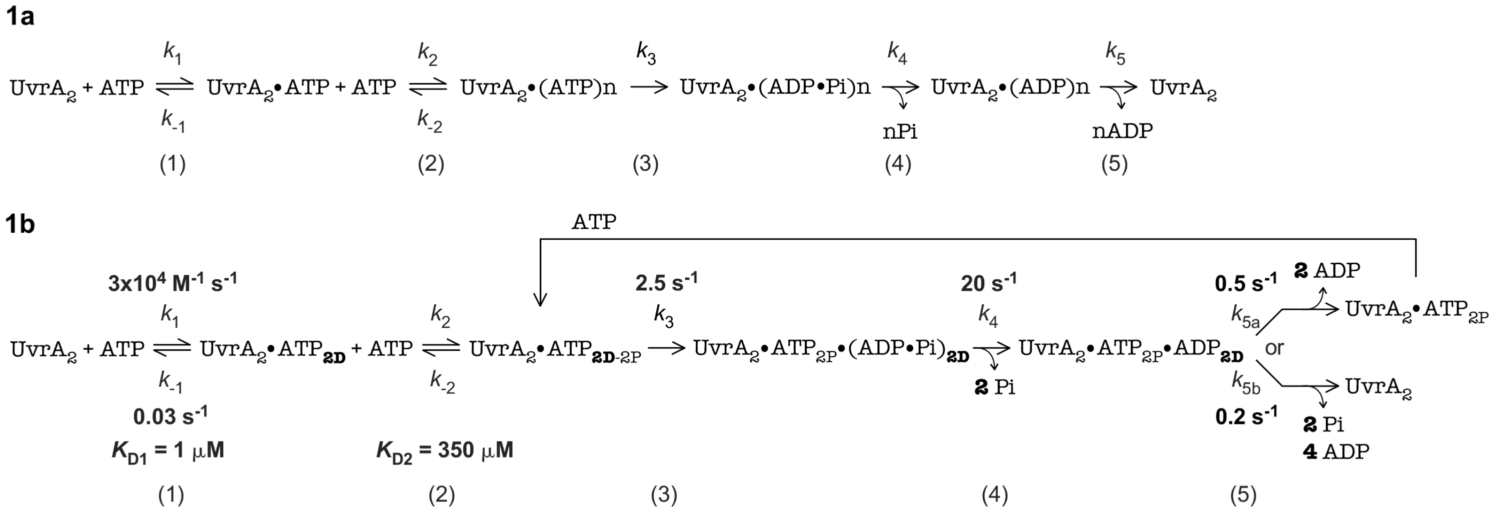
1a
UvrA2 + ATP ⇌ UvrA2•ATP + ATP ⇌ UvrA2•(ATP)n → UvrA2•(ADP•Pi)n → UvrA2•(ADP)n → UvrA2
k1
k-1
k2
k-2
k3
k4
k5
nPi
nADP
(1)
(2)
(3)
(4)
(5)
1b
ATP
3x10^4 M-1 s-1
0.03 s-1
KD1 = 1 μM
KD2 = 350 μM
2.5 s-1
20 s-1
0.5 s-1
0.2 s-1
UvrA2 + ATP ⇌ UvrA2•ATP2D + ATP ⇌ UvrA2•ATP2D-2P → UvrA2•ATP2P•(ADP•Pi)2D → UvrA2•ATP2P•ADP2D
2 Pi
k5a
k5b
2 ADP
UvrA2•ATP2P
or
UvrA2
2 Pi
4 ADP

Figure S5. ATP binding at distal sites, but not ADP, stimulates proximal site ATPase activity. Pre-steady state kinetics of Pi release were measured by mixing $UvrA_2$, -/+ ADP and ^{MDCC}PBP reporter with ATP on a stopped-flow (final concentrations: 0.25 µM $UvrA_2$, 0 - 10 µM ADP, 1 mM ATP and 15 µM ^{MDCC}PBP). **(A)** For wild type $UvrA_2$, the burst is lost at low ADP concentrations sufficient to occupy the distal but not proximal sites, confirming that distal sites catalyze rapid ATP hydrolysis and Pi release in the absence of DNA. Also, the slow steady state rate indicates low level proximal ATPase activity when the distal sites are ADP-bound. **(B)** As seen in Figure 5, the distal site Walker B mutant $^{E854A}UvrA_2$ exhibits a lag followed by a stimulated ATPase rate, indicating that nucleotide binding to distal sites stimulates proximal site activity (the distal site Walker A mutant $^{K643A}UvrA_2$ has low ATPase activity and is shown for comparison). Preincubation of $^{E854A}UvrA_2$ with ADP lengthens the lag phase followed by recovery of the stimulated rate, indicating that the ADP pre-bound at distal sites must be replaced by ATP to stimulate proximal site activity.

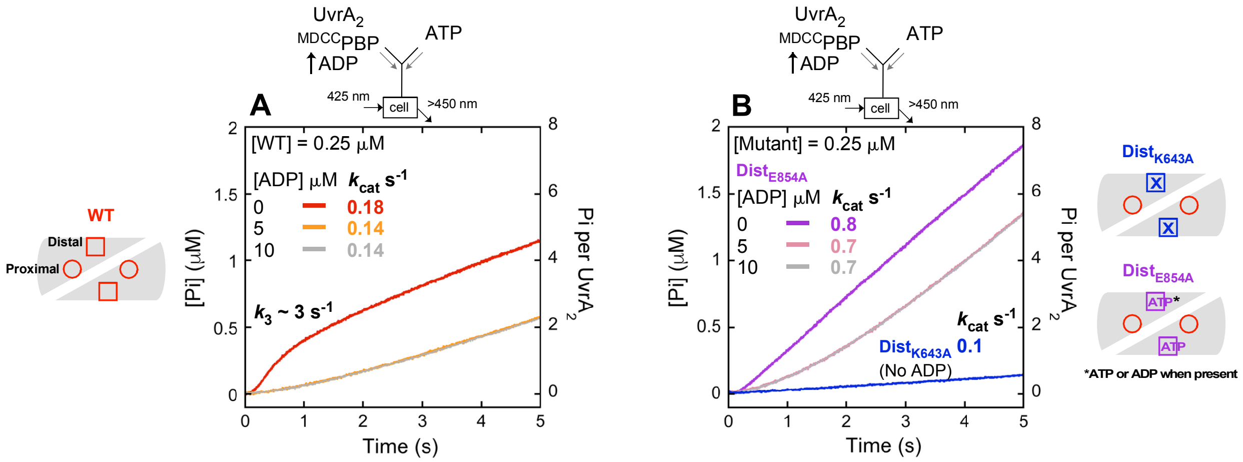

Figure S6. Interaction between $UvrA_2$ and DNA. Binding of **(A)** Fluorescein mid-labeled lesion DNA and **(B)** 6-FAM 5′ end-labeled undamaged (native) DNA to $UvrA_2$ was measured under equilibrium conditions (final concentrations: 0 - 250 nM $UvrA_2$, 7.5 nM DNA). Fluorescence anisotropy of the DNAs increases with $UvrA_2$ concentration, and the resulting binding isotherms show that $UvrA_2$ binds the lesion with 4-fold higher affinity than native DNA ($K_D = 12 \pm 2$ nM versus 45 ± 7 nM, respectively); error bars report standard error of the mean (N = 3).

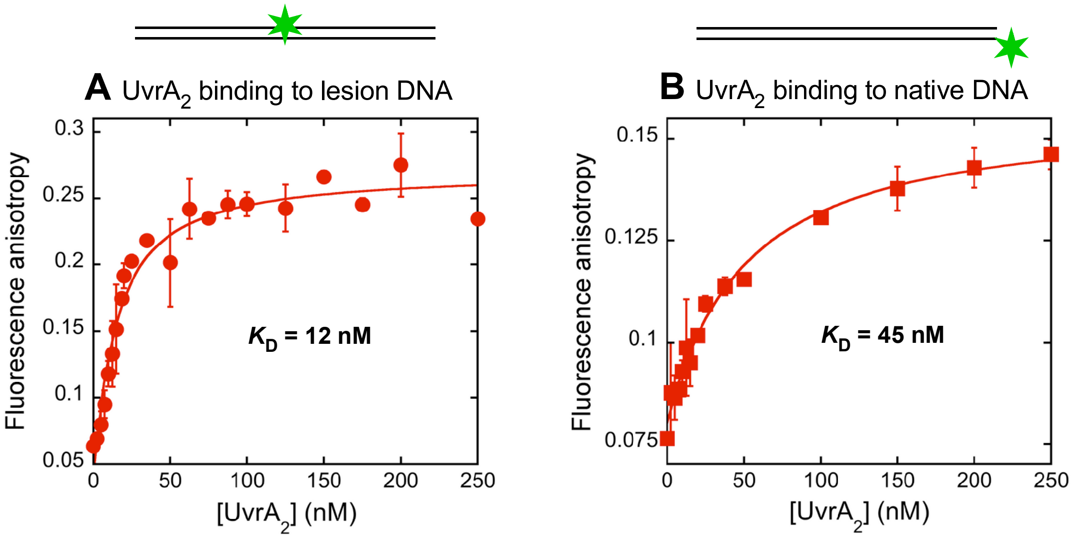

Figure S7. Interaction between $UvrA_2$ and DNA does not alter nucleotide binding. Nucleotide binding kinetics were measured by increase in mant fluorescence over time on mixing $UvrA_2$ with **(A)** mant-ATP or **(B)** mant-ADP in the presence of undamaged or lesion DNA (final concentrations: 0.1 μM $UvrA_2$, 0.2 μM DNA, 10 μM mant-ATP or mant-ADP). The binding rates for are comparable to those in the absence of DNA (see Figure S2 and Table 1).

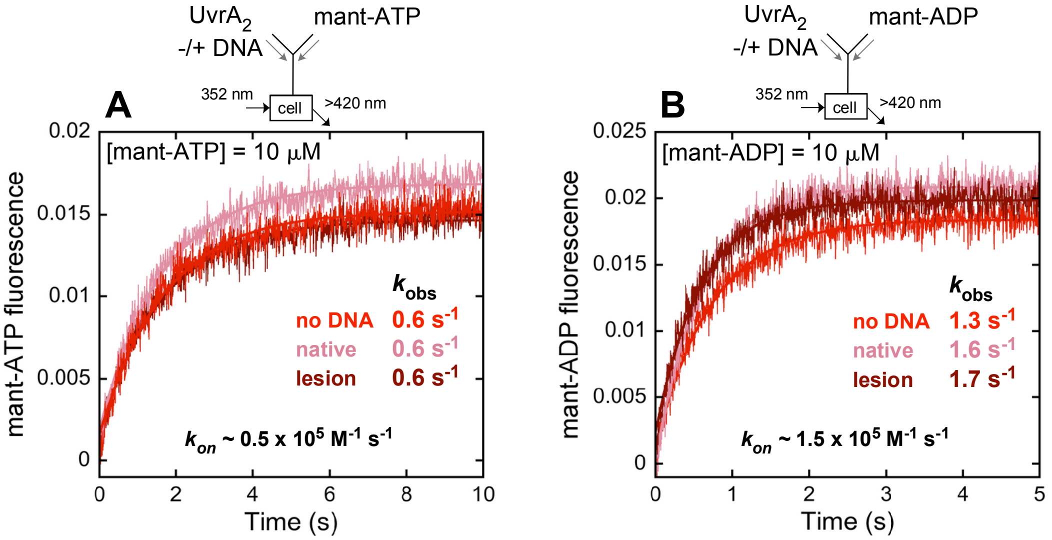

	T. maritima UvrA Δ117-399 (molecular replacement)
**Data collection**	
Space group	C 1 2 1
Cell dimensions	
a, b, c (Å)	143.134, 81.379, 90.416
α, β, γ (°)	90.000, 125.301, 90.000
Wavelength	0.97946
Highest resolution shell	2.082 - 2.01
<I/sig> all data (highest resolution shell)	8.42 (1.09)
Completeness (%) (highest resolution shell)	91.81 (81.93)
Average redundancy (highest resolution shell)	3.9 (3.5)
**Refinement**	
Resolution (Å)	2.01 (CC1/2=0.525)
No. reflections (highest resolution shell)	51809 (4575)
R_{work}/R_{free}	0.1785/0.2079
Number of residues	625
No. atoms	
Protein	4881
Ligands	55
Water	463
B-factors	
Protein	40.57
Ligands	32.87
Water	41.59
Average	40.58
R.M.S. deviations	
Bond lengths (Å)	0.007
Bond angles (°)	1.219
**Ramachandran plot**	
Favored	95.17
Allowed	4.51
Outliers	0.32
**PDB code**	6N9L

Table S1. Data collection and refinement statistics. Resolution was determined using CC0.5>0.5. Density for residues 61-68 was missing.

Organism	PDB	Proximal site nucleotide	Proximal Walker A residues aligned	Proximal RMSD (Å)	Distal site nucleotide	Distal Walker A residues aligned	Distal RMSD (Å)
G. stearothermophilus	2R6F	ADP	31-39	Target	ADP	637-645	Target
G. stearothermophilus	3UWX	None	31-39	0.232	None	637-645	0.141
G. stearothermophilus	3UX8	None	31-39	0.346	ADP	637-645	0.235
T. maritima	3PIH	PPV	31-39	0.603	PPV	617-625	0.222
T. maritima	6N9L	ADP	31-39	0.213	ADP	617-625	0.220
M. tuberculosis	3ZQJ	None	32-40	0.393	None	654-662	0.288
D. radiodurans	2VF7	ADP	43-51	0.256	ADP	530-538	0.371
D. radiodurans	2VF8	ADP	43-51	0.288	ADP	530-538	0.430

Table S2. ATPase site alignment of available UvrA structures. All structures were aligned using the Walker A motif of 2R6F as the target and the "align" function in PyMol. The resulting RMSD is reported for both proximal and distal site Walker A motifs.

REFERENCES

1. Croteau, D.L., DellaVecchia, M.J., Perera, L. and Van Houten, B. (2008) Cooperative damage recognition by UvrA and UvrB: identification of UvrA residues that mediate DNA binding. *DNA Repair (Amst)*, **7**, 392-404.
2. Johnson, K.A. (2009) Fitting enzyme kinetic data with KinTek Global Kinetic Explorer. *Methods Enzymol*, **467**, 601-626.
3. Johnson, K.A., Simpson, Z.B. and Blom, T. (2009) Global kinetic explorer: a new computer program for dynamic simulation and fitting of kinetic data. *Anal Biochem*, **387**, 20-29.
4. Johnson, K.A. (1992) Transient-state kinetic analysis of enzyme reaction pathways. *The Enzymes*, **20**, 1-61.
5. Johnson, K.A., Simpson, Z.B. and Blom, T. (2009) FitSpace explorer: an algorithm to evaluate multidimensional parameter space in fitting kinetic data. *Anal Biochem*, **387**, 30-41.
6. Pakotiprapha, D., Samuels, M., Shen, K., Hu, J.H. and Jeruzalmi, D. (2012) Structure and mechanism of the UvrA-UvrB DNA damage sensor. *Nat Struct Mol Biol*, **19**, 291-298.
7. Sievers, F., Wilm, A., Dineen, D., Gibson, T.J., Karplus, K., Li, W., Lopez, R., McWilliam, H., Remmert, M., Söding, J. *et al.* (2011) Fast, scalable generation of high-quality protein multiple sequence alignments using Clustal Omega. *Mol Syst Biol*, **7**, Article number 539 doi:510.1038/msb.2011.1075.
8. Jaciuk, M., Nowak, E., Skowronek, K., Tanska, A. and Nowotny, M. (2011) Structure of UvrA nucleotide excision repair protein in complex with modified DNA. *Nat Struct Mol Biol*, **18**, 191-197.
9. Otwinowski, Z. and Minor, W. (1997) Processing of X-ray diffraction data collected in oscillation mode. *Methods Enzymol*, **276**, 307-326.
10. Adams, P.D., Baker, D., Brunger, A.T., Das, R., DiMaio, F., Read, R.J., Richardson, D.C., Richardson, J.S. and Terwilliger, T.C. (2013) Advances, interactions, and future developments in the CNS, Phenix, and Rosetta structural biology software systems. *Annu Rev Biophys*, **42**, 265-287.
11. Echols, N., Moriarty, N.W., Klei, H.E., Afonine, P.V., Bunkóczi, G., Headd, J.J., McCoy, A.J., Oeffner, R.D., Read, R.J., Terwilliger, T.C. *et al.* (2014) Automating crystallographic structure solution and refinement of protein-ligand complexes. *Acta Crystallogr D Biol Crystallogr*, **70**, 144-154.
12. Headd, J.J., Echols, N., Afonine, P.V., Moriarty, N.W., Gildea, R.J. and Adams, P.D. (2014) Flexible torsion-angle noncrystallographic symmetry restraints for improved macromolecular structure refinement. *Acta Crystallogr D Biol Crystallogr*, **70**, 1346-1356.
13. Emsley, P., Lohkamp, B., Scott, W.G. and Cowtan, K. (2010) Features and development of Coot. *Acta Crystallogr D Biol Crystallogr*, **66**, 486-501.
14. De Castro, E., Sigrist, C.J.A., Gattiker, A., Bulliard, V., Langendijk-Genevaux, P.S., Gasteiger, E., Bairoch, A. and Hulo, N. (2006) ScanProsite: detection of PROSITE signature matches and ProRule-associated functional and structural residues in proteins. *Nucleic Acids Res*, **34(Web Server Issue)**, W362-365.

15. Hayward, S. and Berendsen, H.J.C. (1998) Systematic Analysis of Domain Motions in Proteins from Conformational Change: New Results on Citrate Synthase and T4 Lysozyme. *Proteins: Structure, Function, and Bioinformatics*, **30**, 144-154.
16. Pakotiprapha, D., Inuzuka, Y., Bowman, B.R., Moolenaar, G.F., Goosen, N., Jeruzalmi, D. and Verdine, G.L. (2008) Crystal structure of Bacillus stearothermophilus UvrA provides insight into ATP-modulated dimerization, UvrB interaction, and DNA binding. *Mol Cell*, **29**, 122-133.
